# Supplementary material for: H4K20me3 is important for Ash1-mediated H3K36me3 and transcriptional silencing in facultative heterochromatin in a fungal pathogen
Source: PLoS Genet. 2023 Sep 25;19(9):e1010945. doi: 10.1371/journal.pgen.1010945 (PMC10553808; doi:10.1371/journal.pgen.1010945)
Supplement: S1 Fig — A) Alignment of SET domains of H4K20 methyltransferases in different species. SET domain of human EZH2 is used as an outgroup. B) Phylogenetic tree based on the alignment in A. Numbers indicate substitutions per site. C) Accession numbers of proteins used for analyses shown in A and B. (PDF) [file pgen.1010945.s012.pdf]

A

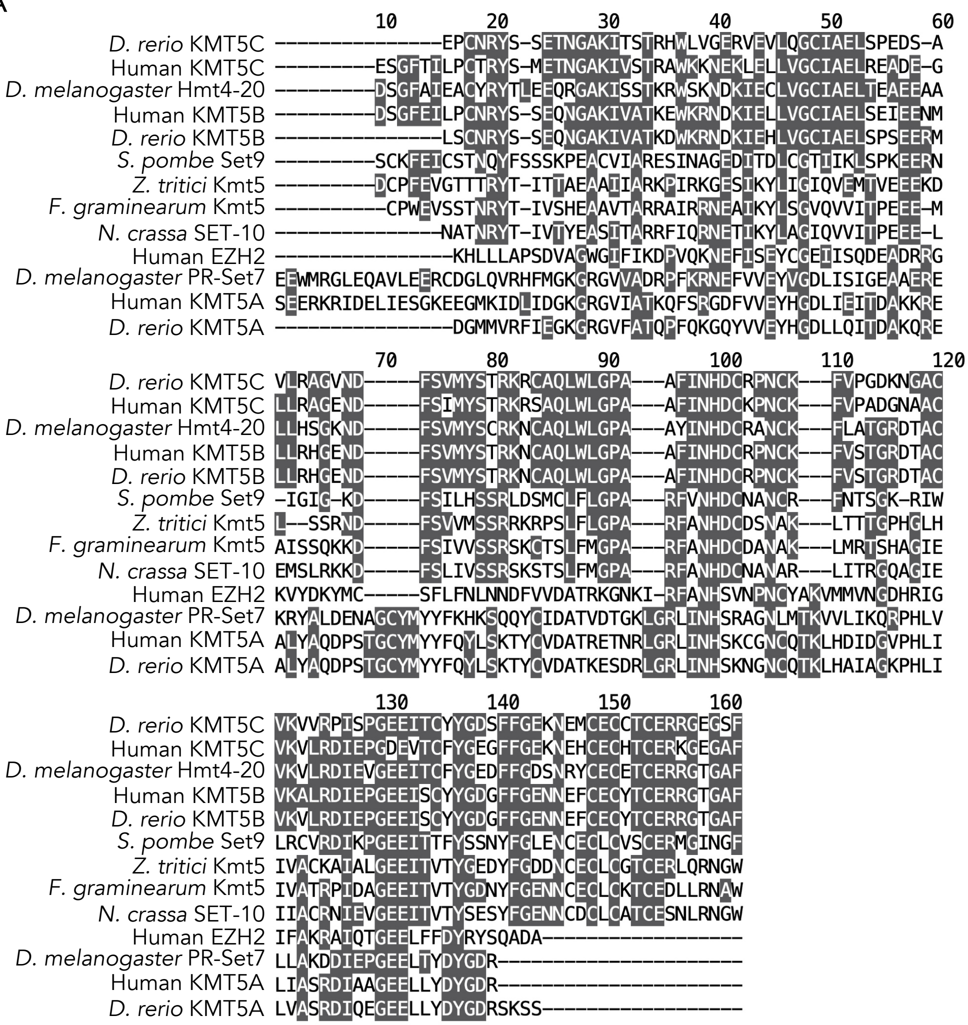

B

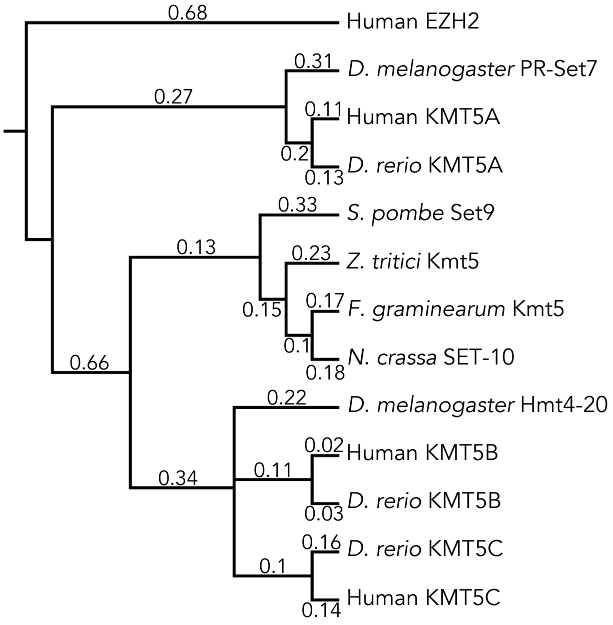

C

**Protein**	**Accession**
Human EZH2	XP_005250019.1
D. melanogaster PR-Set7	NP_001247100.1
Human KMT5A	Q9NQR1.3
D. rerio KMT5A	NP_001038814.2
S. pombe Set9	NP_588078.1
Z. tritici Kmt5	SMQ48785.1
F. graminearum Kmt5	XP_011326140.1
N. crassa SET-10	XP_963033.2
D. melanogaster Hmt4-20	NP_569853.1
Human KMT5B	NP_001356355.1
D. rerio KMT5B	Q5U3H2.2
D. rerio KMT5C	NP_001091656.1
Human KMT5C	NP_116090.2

S1 Fig. Comparison of H4K20 HMT SET domains. A) Alignment of SET domains of H4K20 methyltransferases in different species. SET domain of human EZH2 is used as an outgroup. B) Phylogenetic tree based on the alignment in A. Numbers indicate substitutions per site. C) Accession numbers of proteins used for analyses shown in A and B.
