# Supplementary material for: H4K20me3 is important for Ash1-mediated H3K36me3 and transcriptional silencing in facultative heterochromatin in a fungal pathogen
Source: PLoS Genet. 2023 Sep 25;19(9):e1010945. doi: 10.1371/journal.pgen.1010945 (PMC10553808; doi:10.1371/journal.pgen.1010945)
Supplement: S2 Fig — (PDF) [file pgen.1010945.s013.pdf]

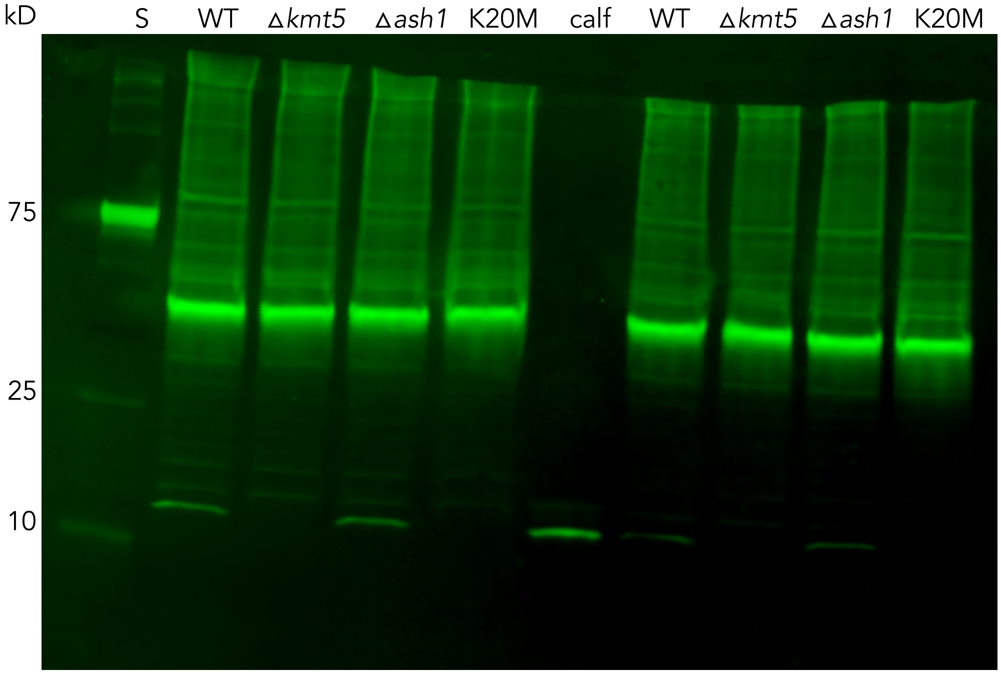

αH4K20me3 (Active motif 39180, pAb, anti-rabbit, green) 1:2000
IRDye 800CW Goat anti-Rabbit IgG (LI-COR 926-32211) 1:5000
Standard: BioRad precision plus protein kaleidoscope pre-stained protein

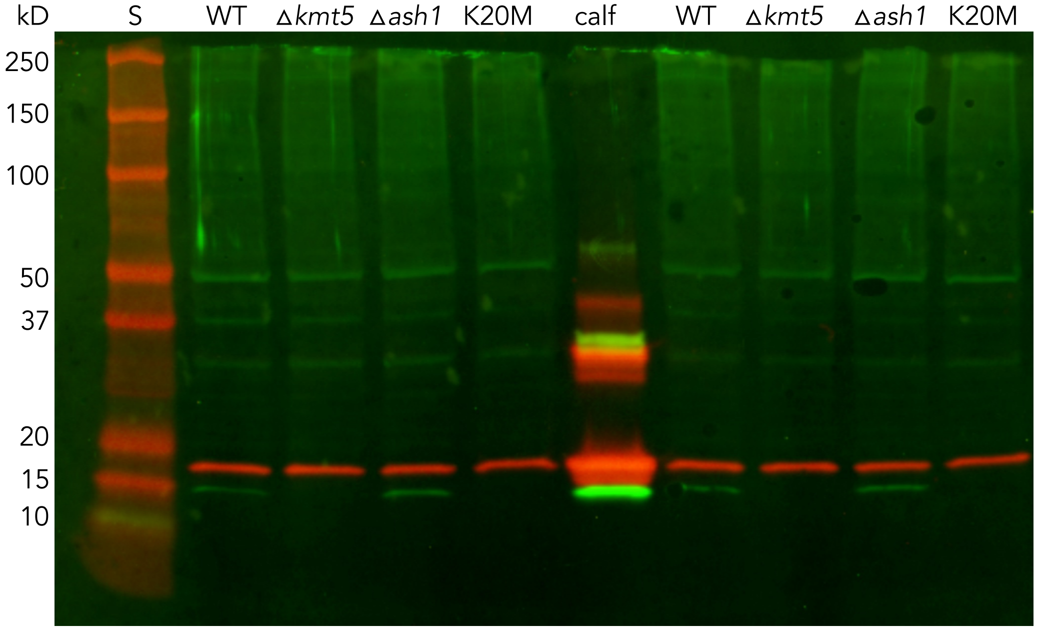

αH3 (Active motif 39763, mAb, anti-mouse, red) 1:5000
αH4K20me1 (abcam 9051, pAb, anti-rabbit, green) 1:1000
IRDye 680RD Goat anti-Mouse IgG (LI-COR 926-68070) 1:5000
IRDye 800CW Goat anti-Rabbit IgG (LI-COR 926-32211) 1:5000
Standard: BioRad precision plus protein kaleidoscope pre-stained protein

S2 Fig. Uncropped western blots for detection of H4K20me1, H4K20me3, and H3.
