# Supplementary material for: H4K20me3 is important for Ash1-mediated H3K36me3 and transcriptional silencing in facultative heterochromatin in a fungal pathogen
Source: PLoS Genet. 2023 Sep 25;19(9):e1010945. doi: 10.1371/journal.pgen.1010945 (PMC10553808; doi:10.1371/journal.pgen.1010945)
Supplement: S3 Fig — A) ∆kmt5 mutants show more bands in addition to the expected bands. Because one of the flanks used for homologous recombination and as a probe for Southern analyses is a repetitive element, integration into the repetitive element may have altered restriction sites, and additional bands are detected because of TE copy number. Deletion of kmt5 was confirmed in all relevant ChIP- and RNA-seq datasets. Strains used for further analyses were 95 and 100. B) Confirmation of ∆ash1. Strains used for further analyses were 5 and 25. Deletion of ash1 was confirmed in all relevant ChIP- and RNA-seq datasets. (PDF) [file pgen.1010945.s014.pdf]

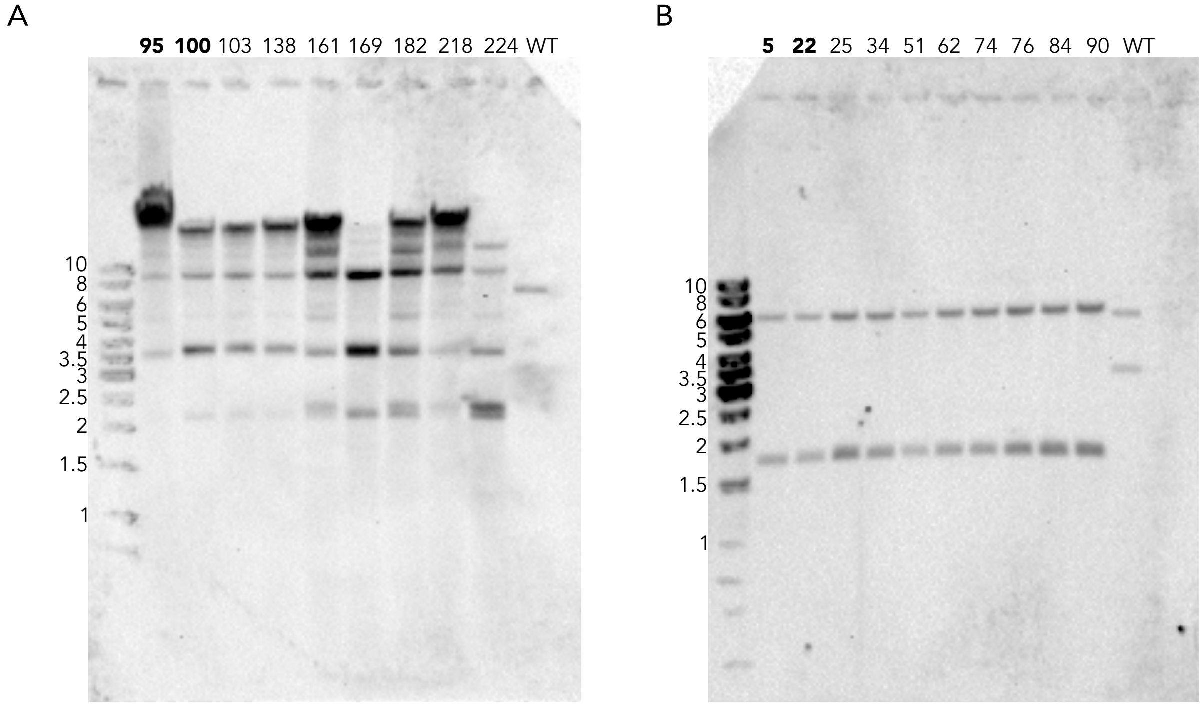

A)

Enzyme:	Expected bands:	
NdeI *SphI*	WT	6626 bp
	Δ*kmt5*	3443 bp
		2059 bp
		132 bp

B)

Enzyme:	Expected bands:	
PvuI	WT	5819 bp
		3346 bp
	Δ*ash1*	6219 bp
		1746 bp

S3 Fig. Southern blots to confirm deletion of *kmt5* and *ash1*. A) Δ*kmt5* mutants show more bands in addition to the expected bands. Because one of the flanks used for homologous recombination and as a probe for Southern analyses is a repetitive element, integration into the repetitive element may have altered restriction sites, and additional bands are detected because of TE copy number. Deletion of *kmt5* was confirmed in all relevant ChIP- and RNA-seq datasets. Strains used for further analyses were 95 and 100. B) Confirmation of Δ*ash1*. Strains used for further analyses were 5 and 25. Deletion of *ash1* was confirmed in all relevant ChIP- and RNA-seq datasets.
