# Supplementary material for: H4K20me3 is important for Ash1-mediated H3K36me3 and transcriptional silencing in facultative heterochromatin in a fungal pathogen
Source: PLoS Genet. 2023 Sep 25;19(9):e1010945. doi: 10.1371/journal.pgen.1010945 (PMC10553808; doi:10.1371/journal.pgen.1010945)
Supplement: S4 Fig — (PDF) [file pgen.1010945.s015.pdf]

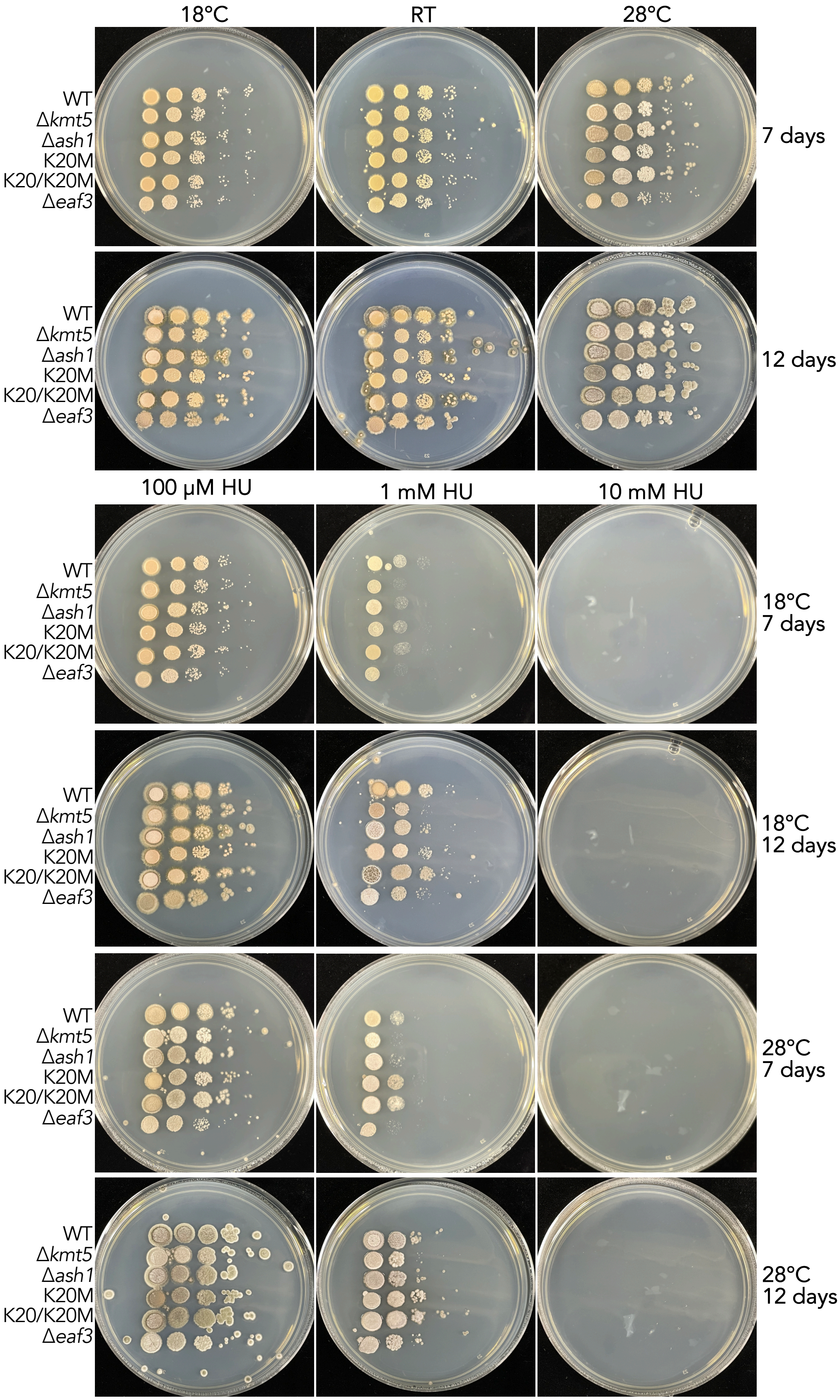
18°C
RT
28°C
WT
Δkmt5
Δash1
K20M
K20/K20M
Δeaf3
7 days
12 days
100 μM HU
1 mM HU
10 mM HU
18°C
7 days
18°C
12 days
28°C
7 days
28°C
12 days

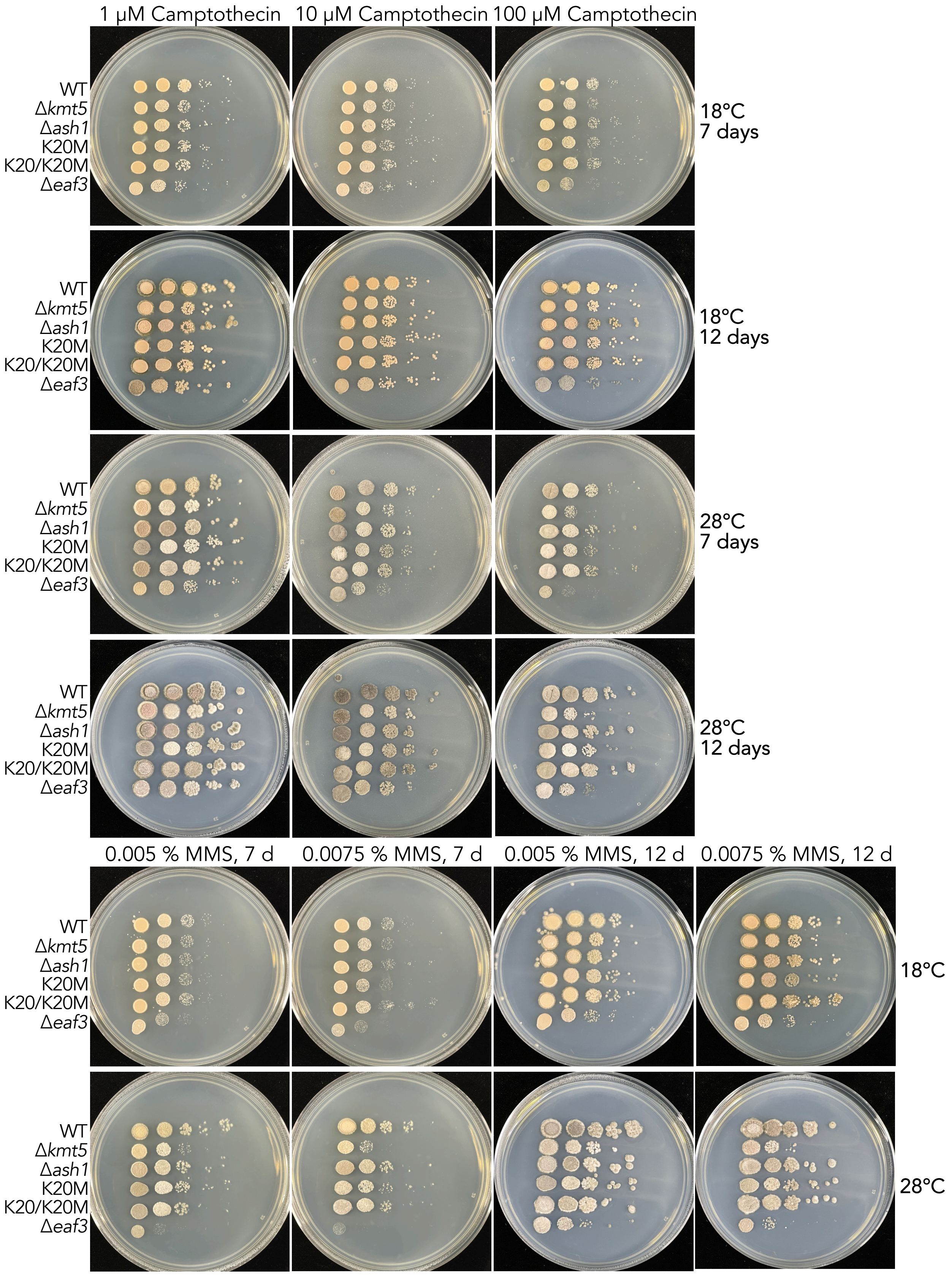
1 µM Camptothecin
10 µM Camptothecin
100 µM Camptothecin
WT
Δkmt5
Δash1
K20M
K20/K20M
Δeaf3
18°C
7 days
18°C
12 days
28°C
7 days
28°C
12 days
0.005 % MMS, 7 d
0.0075 % MMS, 7 d
0.005 % MMS, 12 d
0.0075 % MMS, 12 d
18°C
28°C

S4 Fig. Phenotypic characterization of wild type (WT), Δ*kmt5*, Δ*ash1*, K20M, K20/K20M, and Δ*eaf3* under different genotoxic stress conditions and temperatures.
