# Supplementary material for: H4K20me3 is important for Ash1-mediated H3K36me3 and transcriptional silencing in facultative heterochromatin in a fungal pathogen
Source: PLoS Genet. 2023 Sep 25;19(9):e1010945. doi: 10.1371/journal.pgen.1010945 (PMC10553808; doi:10.1371/journal.pgen.1010945)
Supplement: S5 Fig — A) ChIP-seq shows wild type-like enrichment of H4K20me3 and H3K36me3 for both, kmt5 and ash1 complementation strains. The ash1 complementation strain is lacking chromosome 16. B) Phenotypic assays performed at 18°C and RT (23°C) testing genotoxic stresses. YMS plates were used as a control. Pictures were taken seven days after inoculation. Complementation strains exhibit wild-type growth. (PDF) [file pgen.1010945.s016.pdf]

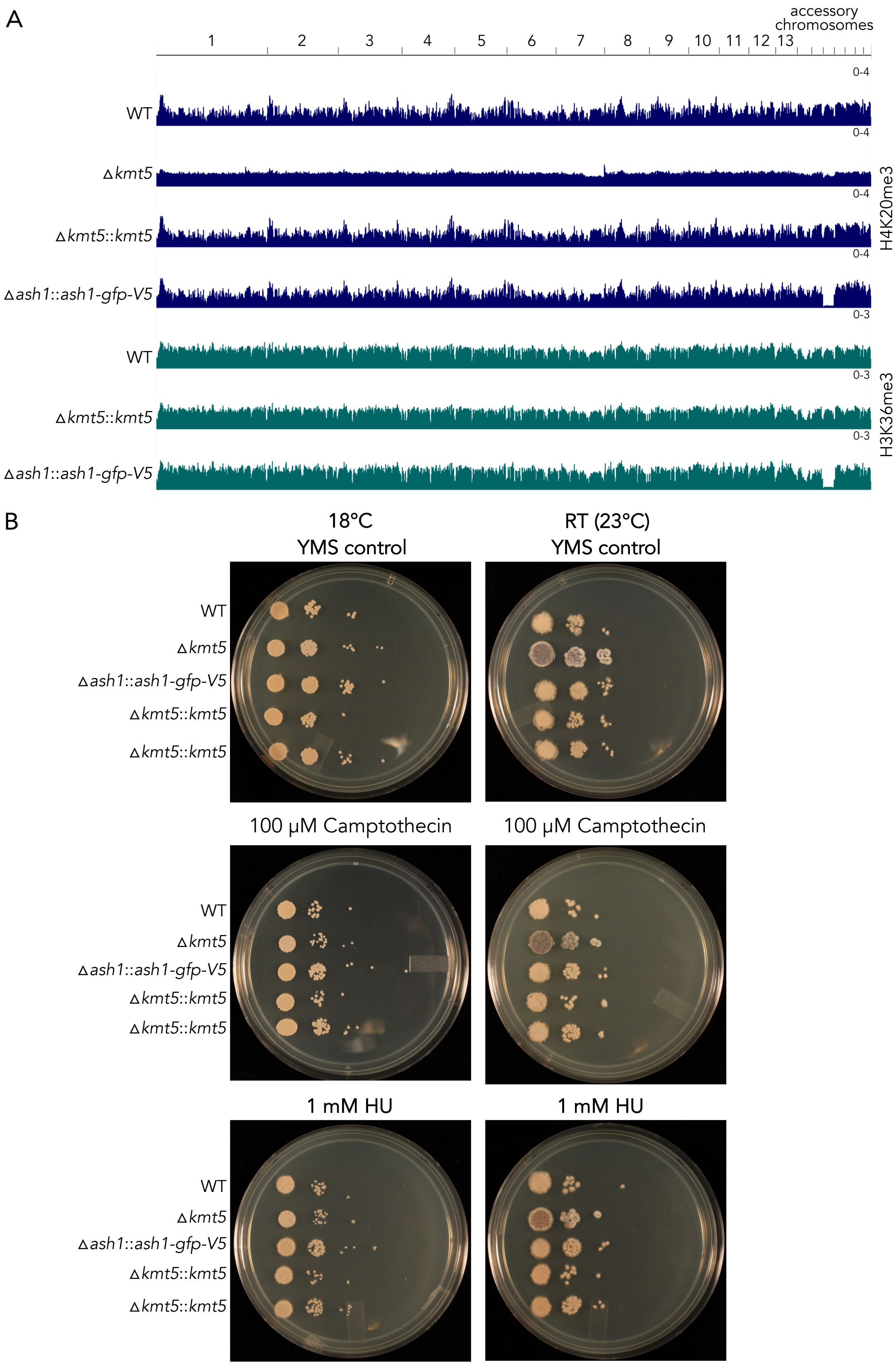
A
accessory chromosomes
1
2
3
4
5
6
7
8
9
10
11
12
13
0-4
WT
Δkmt5
Δkmt5::kmt5
Δash1::ash1-gfp-V5
H4K20me3
0-3
WT
Δkmt5::kmt5
Δash1::ash1-gfp-V5
H3K36me3
B
18°C YMS control
RT (23°C) YMS control
WT
Δkmt5
Δash1::ash1-gfp-V5
Δkmt5::kmt5
Δkmt5::kmt5
100 µM Camptothecin
100 µM Camptothecin
1 mM HU
1 mM HU

S5 Fig. Analysis of *kmt5* and *ash1* complementation strains. A) ChIP-seq shows wild type-like enrichment of H4K20me3 and H3K36me3 for both, *kmt5* and *ash1* complementation strains. The *ash1* complementation strain is lacking chromosome 16. B) Phenotypic assays performed at 18°C and RT (23°C) testing genotoxic stresses. YMS plates were used as a control. Pictures were taken seven days after inoculation. Complementation strains exhibit wild-type growth.
