# Supplementary material for: H4K20me3 is important for Ash1-mediated H3K36me3 and transcriptional silencing in facultative heterochromatin in a fungal pathogen
Source: PLoS Genet. 2023 Sep 25;19(9):e1010945. doi: 10.1371/journal.pgen.1010945 (PMC10553808; doi:10.1371/journal.pgen.1010945)
Supplement: S6 Fig — (PDF) [file pgen.1010945.s017.pdf]

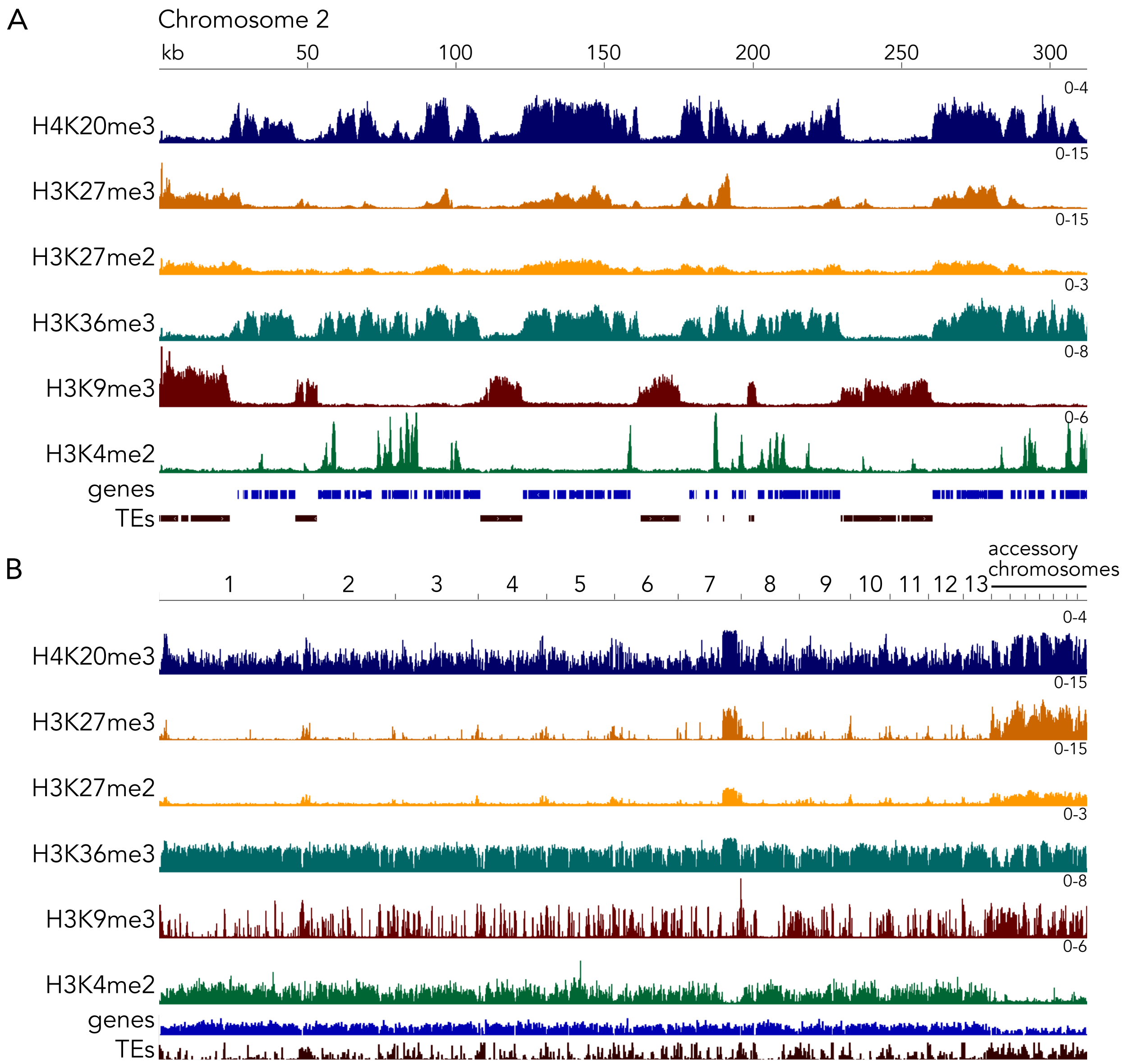

S6 Fig. Distribution of H4K20me3, H3K36me3, H3K27me3, H3K9me3, and H3K4me2 in wild type on chromosome 2 as an example region A) and genome wide B).
