# Supplementary material for: H4K20me3 is important for Ash1-mediated H3K36me3 and transcriptional silencing in facultative heterochromatin in a fungal pathogen
Source: PLoS Genet. 2023 Sep 25;19(9):e1010945. doi: 10.1371/journal.pgen.1010945 (PMC10553808; doi:10.1371/journal.pgen.1010945)
Supplement: S7 Fig — While we observed subtle changes outside of facultative heterochromatin, we observed the most prevalent differences in regions of facultative heterochromatin. (PDF) [file pgen.1010945.s018.pdf]

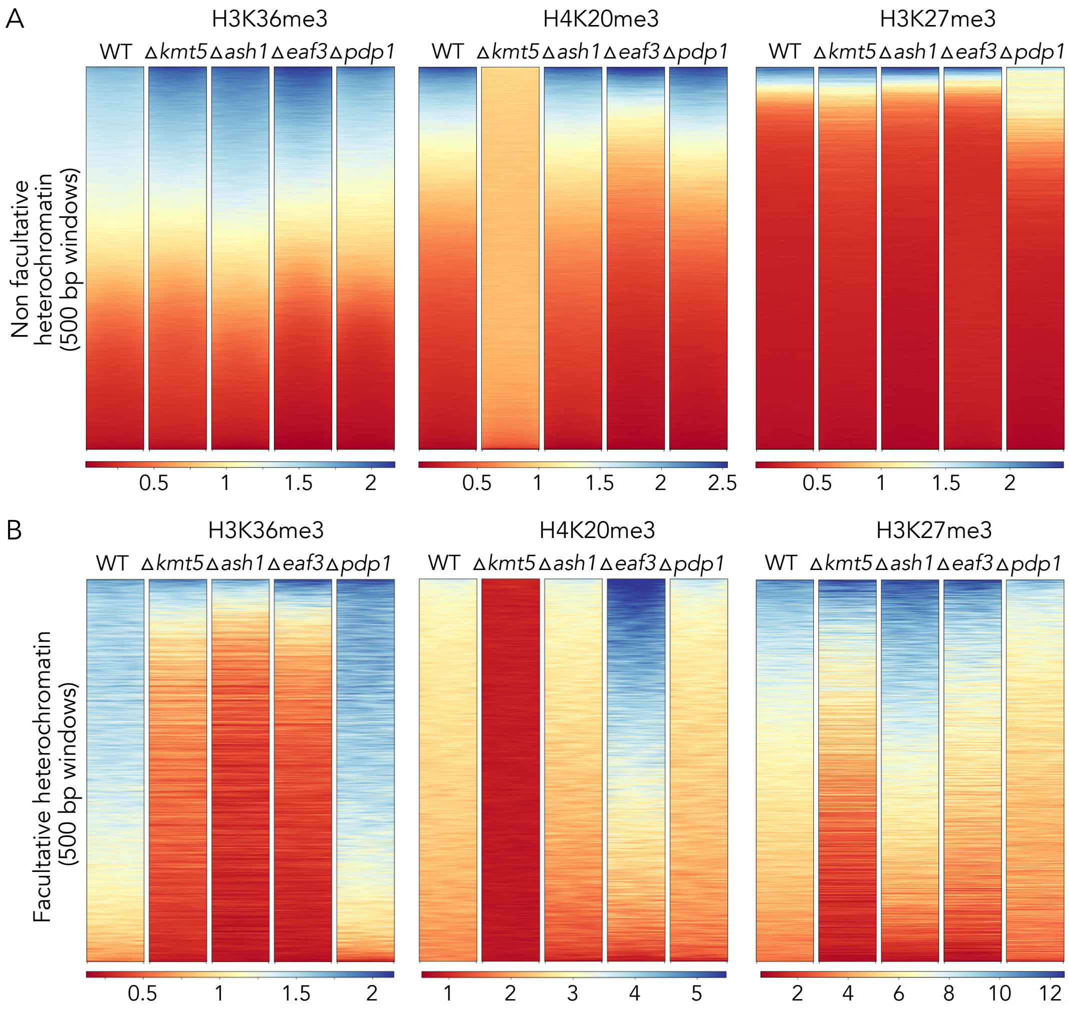

S7 Fig. Enrichment of different histone marks outside A) and within B) facultative heterochromatin regions in all deletion mutants. While we observed subtle changes outside of facultative heterochromatin, we observed the most prevalent differences in regions of facultative heterochromatin.
