# Supplementary material for: H4K20me3 is important for Ash1-mediated H3K36me3 and transcriptional silencing in facultative heterochromatin in a fungal pathogen
Source: PLoS Genet. 2023 Sep 25;19(9):e1010945. doi: 10.1371/journal.pgen.1010945 (PMC10553808; doi:10.1371/journal.pgen.1010945)
Supplement: S8 Fig — There are no ChIP-seq data for H3K36me3 in the tagged Kmt5 strains. 1tagged kmt5 was integrated in the ∆kmt5 strain; 2tagged kmt5 was integrated in the wild-type strain. (PDF) [file pgen.1010945.s019.pdf]

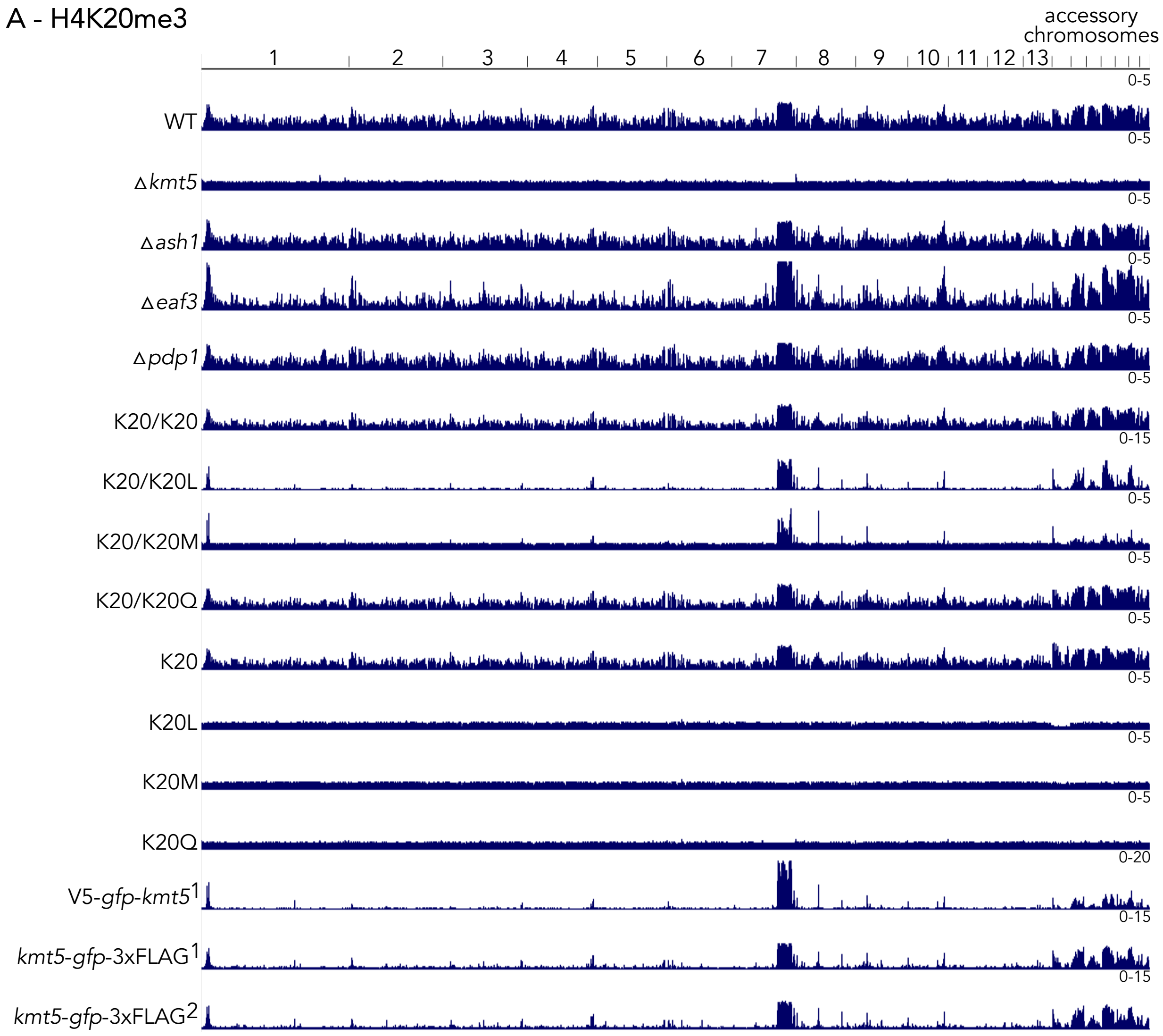
A - H4K20me3
accessory chromosomes
1
2
3
4
5
6
7
8
9
10
11
12
13
0-5
WT
0-5
Δkmt5
0-5
Δash1
0-5
Δeaf3
0-5
Δpdp1
0-5
K20/K20
0-15
K20/K20L
0-5
K20/K20M
0-5
K20/K20Q
0-5
K20
0-5
K20L
0-5
K20M
0-5
K20Q
0-20
V5-gfp-kmt5[1]
0-15
kmt5-gfp-3xFLAG[1]
0-15
kmt5-gfp-3xFLAG[2]

B - H3K27me3

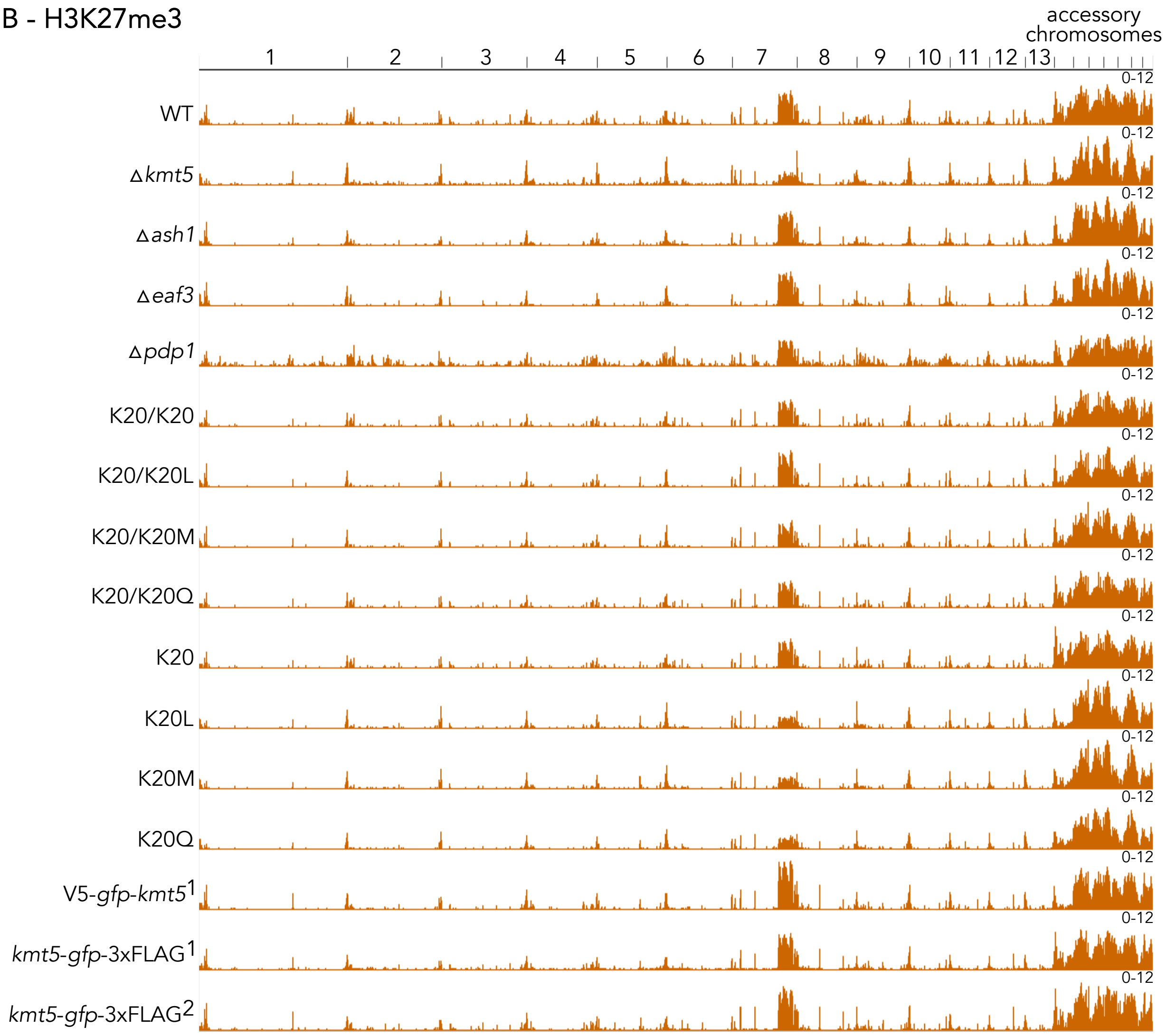
accessory chromosomes
1
2
3
4
5
6
7
8
9
10
11
12
13
0-12
WT
Δkmt5
Δash1
Δeaf3
Δpdp1
K20/K20
K20/K20L
K20/K20M
K20/K20Q
K20
K20L
K20M
K20Q
V5-gfp-kmt5[1]
kmt5-gfp-3xFLAG[1]
kmt5-gfp-3xFLAG[2]

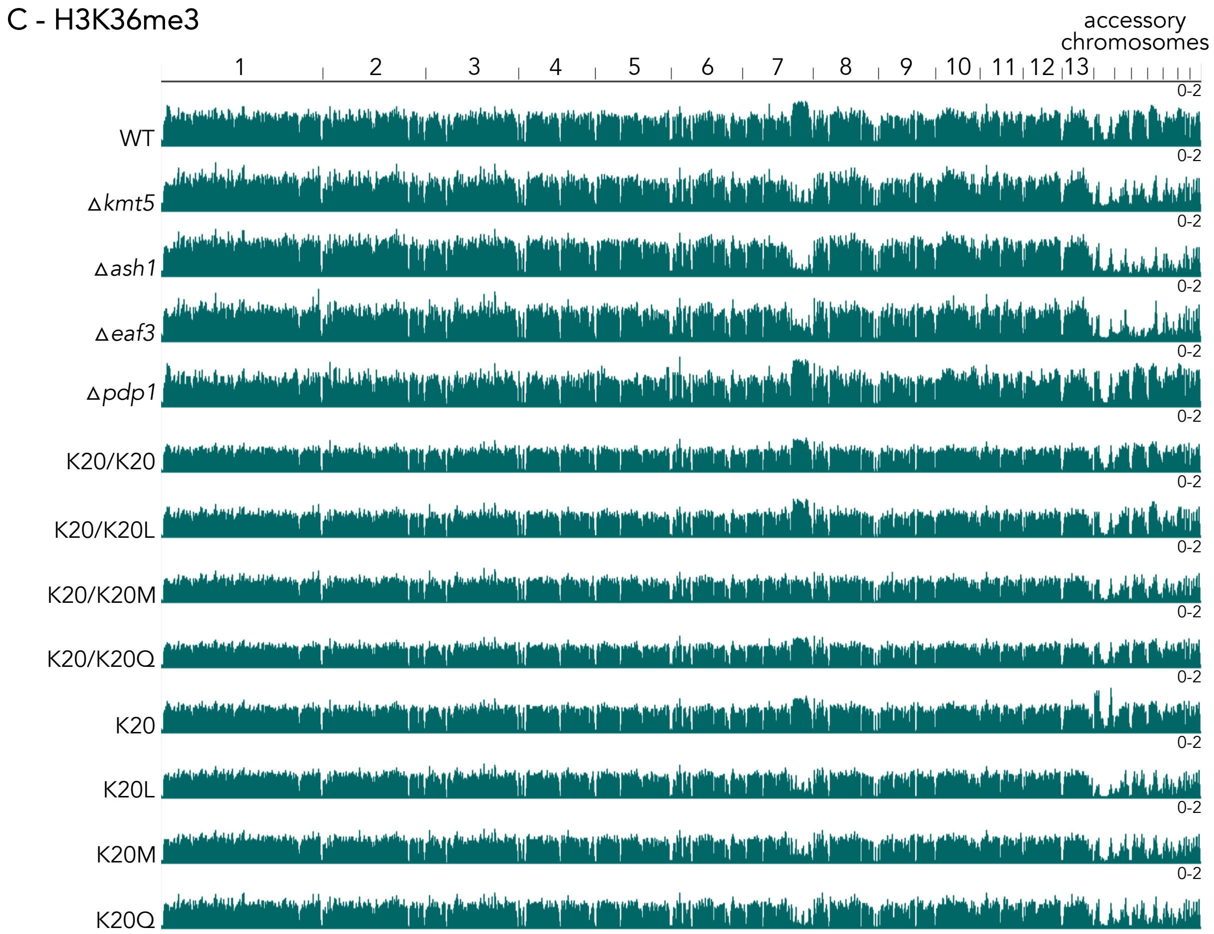

S8 Fig. Genome-wide distribution of A) H4K20me3, B) H3K27me3, and C) H3K36me3 in all mutants generated in this study. There are no ChIP-seq data for H3K36me3 in the tagged Kmt5 strains. [1]tagged *kmt5* was integrated in the Δ*kmt5* strain; [2]tagged *kmt5* was integrated in the wild-type strain.
