# Supplementary material for: H4K20me3 is important for Ash1-mediated H3K36me3 and transcriptional silencing in facultative heterochromatin in a fungal pathogen
Source: PLoS Genet. 2023 Sep 25;19(9):e1010945. doi: 10.1371/journal.pgen.1010945 (PMC10553808; doi:10.1371/journal.pgen.1010945)
Supplement: S9 Fig — A) The ∆set2 mutants grow very slowly. Growth appeared ~ 14 days post inoculation on YMS and incubation at 18°C (compared to ~2 days for wild type) and was limited to sparse hyphal growth, as shown in B) by microscopy. In contrast, the wild type predominantly produces spores under these conditions; ∆set2 mutant seemed unable to produce spores. (PDF) [file pgen.1010945.s020.pdf]

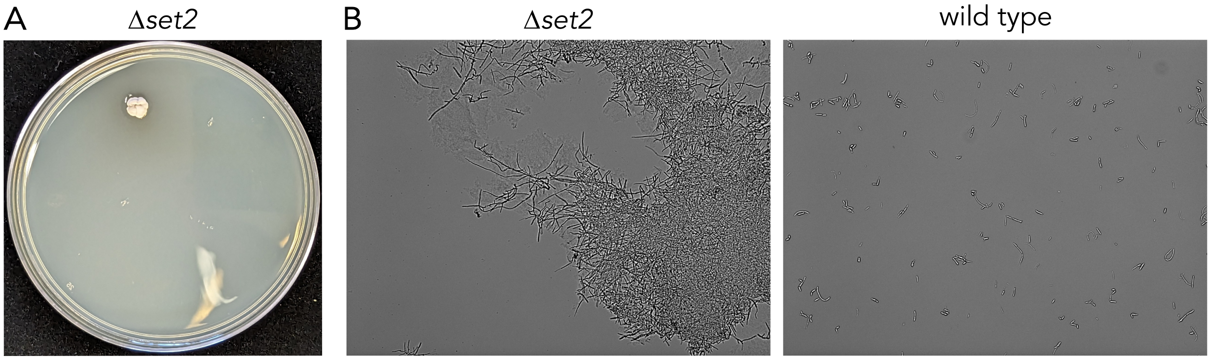

S9 Fig. Phenotype of Δ*set2* mutants. A) The Δ*set2* mutants grow very slowly. Growth appeared ~ 14 days post inoculation on YMS and incubation at 18°C (compared to ~2 days for wild type) and was limited to sparse hyphal growth, as shown in B) by microscopy. In contrast, the wild type predominantly produces spores under these conditions; Δ*set2* mutant seemed unable to produce spores.
