# Supplementary material for: H4K20me3 is important for Ash1-mediated H3K36me3 and transcriptional silencing in facultative heterochromatin in a fungal pathogen
Source: PLoS Genet. 2023 Sep 25;19(9):e1010945. doi: 10.1371/journal.pgen.1010945 (PMC10553808; doi:10.1371/journal.pgen.1010945)
Supplement: S10 Fig — (PDF) [file pgen.1010945.s021.pdf]

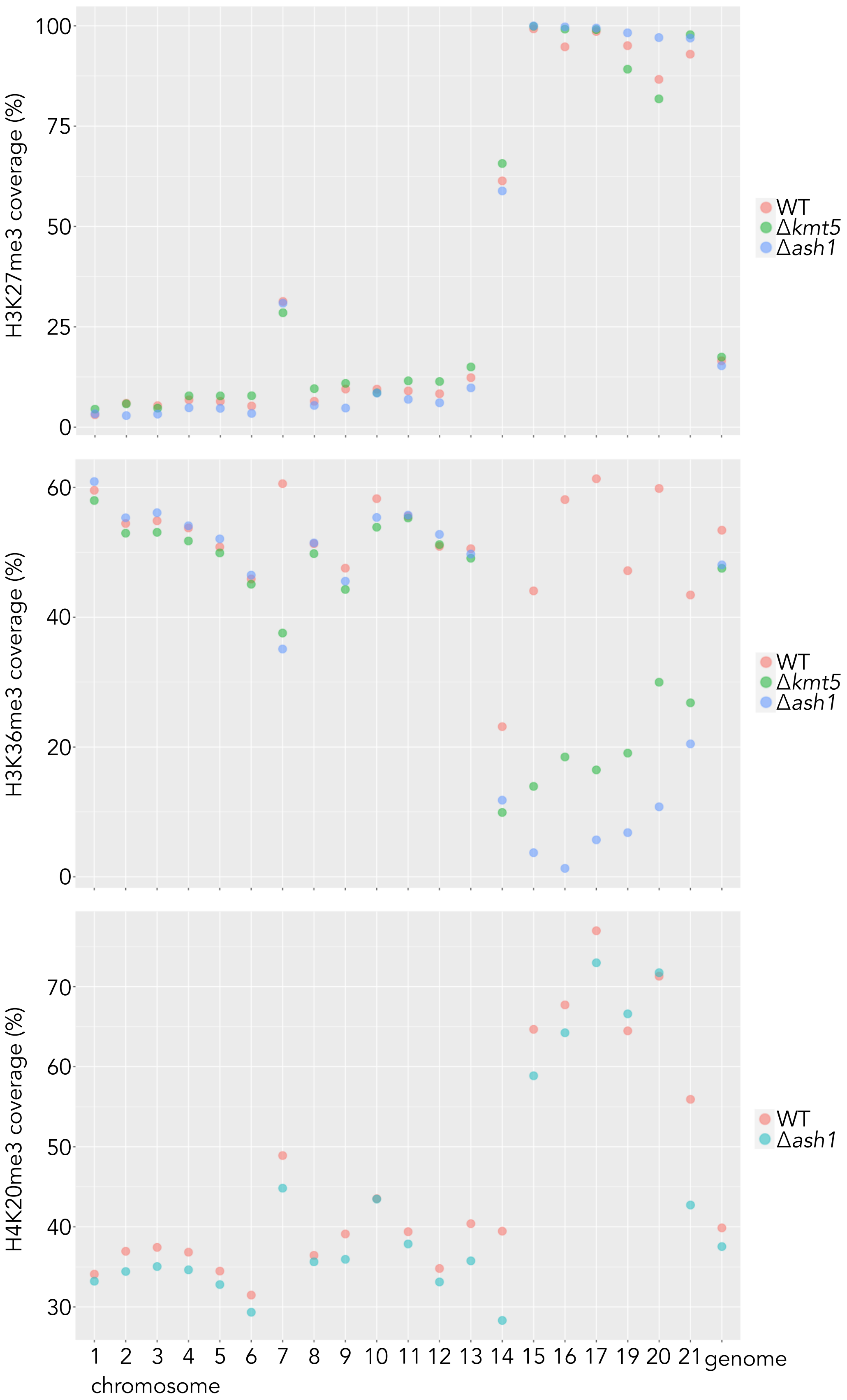
H3K27me3 coverage (%)
H3K36me3 coverage (%)
H4K20me3 coverage (%)
WT
Δkmt5
Δash1
WT
Δkmt5
Δash1
WT
Δash1
100
75
50
25
0
60
40
20
0
70
60
50
40
30
1 2 3 4 5 6 7 8 9 10 11 12 13 14 15 16 17 19 20 21 genome
chromosome

S10 Fig. Percent coverage of bp of histone marks H3K27me3, H3K36me3 and H4K20me3 in wild type, Δ*kmt5* and Δ*ash1* strains. Peaks were called using HOMER (73) and genome coverage of enriched regions was calculated with bedtools genomecov (82)**.**
