# Supplementary material for: H4K20me3 is important for Ash1-mediated H3K36me3 and transcriptional silencing in facultative heterochromatin in a fungal pathogen
Source: PLoS Genet. 2023 Sep 25;19(9):e1010945. doi: 10.1371/journal.pgen.1010945 (PMC10553808; doi:10.1371/journal.pgen.1010945)
Supplement: S11 Fig — A) ChIP-seq tracks (chromosome 10 as an example region) show that H3K27me3 enrichment increases in gene-dense regions that are also enriched with H4K20me3 and H3K36me3 but not H3K9me3. (PDF) [file pgen.1010945.s022.pdf]

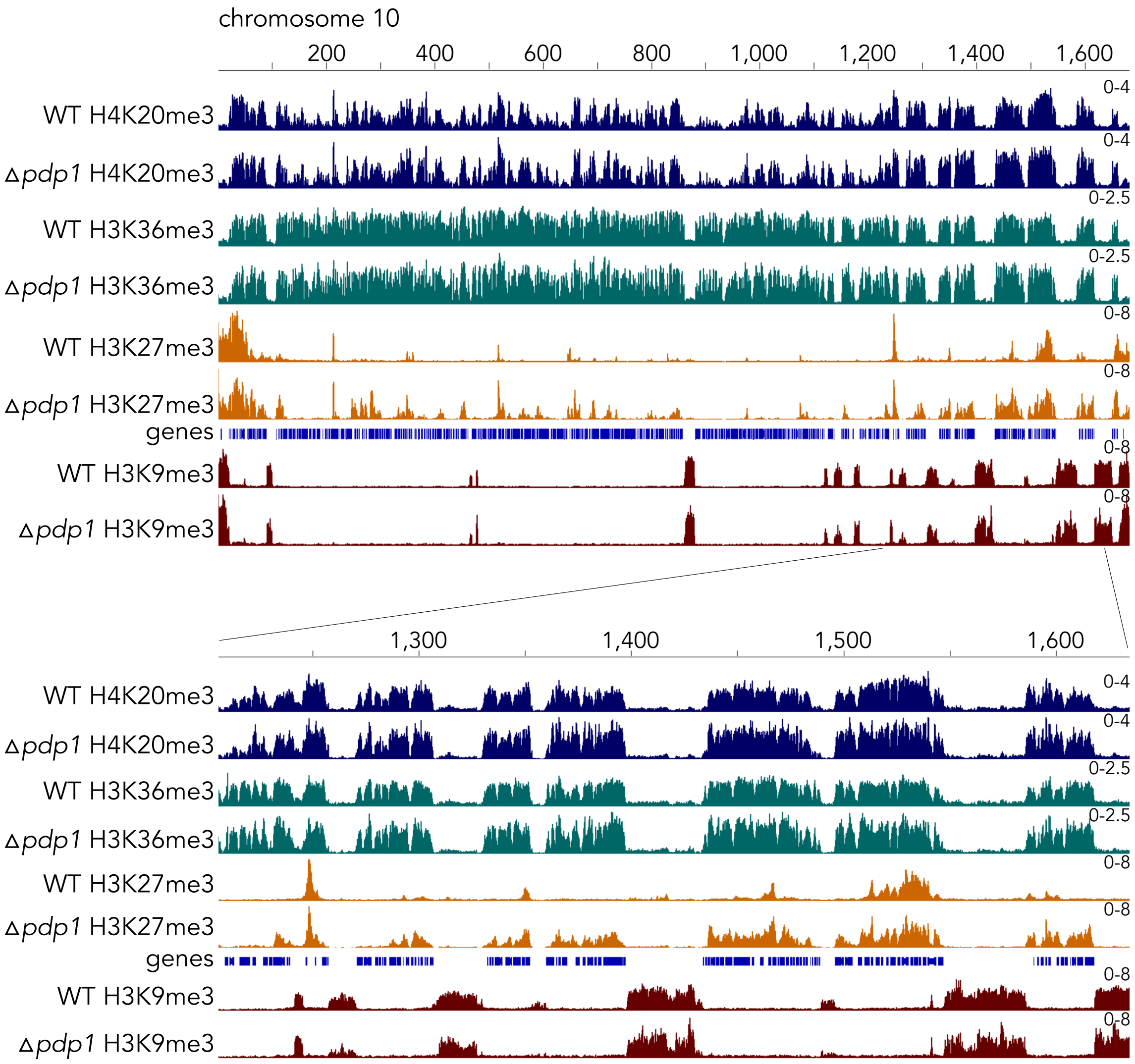

S11 Fig. ChIP-seq of the Δ*pdp1* mutant revealed an increase in H3K27me3 outside of facultative heterochromatin regions in wild type (WT). A) ChIP-seq tracks (chromosome 10 as an example region) show that H3K27me3 enrichment increases in gene-dense regions that are also enriched with H4K20me3 and H3K36me3 but not H3K9me3.
