# Supplementary material for: H4K20me3 is important for Ash1-mediated H3K36me3 and transcriptional silencing in facultative heterochromatin in a fungal pathogen
Source: PLoS Genet. 2023 Sep 25;19(9):e1010945. doi: 10.1371/journal.pgen.1010945 (PMC10553808; doi:10.1371/journal.pgen.1010945)
Supplement: S12 Fig — A) Principal Component Analysis (PCA) plot of all sequenced replicate strains. B) Venn diagram of upregulated genes (DESeq2, padj ≤0.01, | log2 fold change | ≥0.585) in all mutants compared to wild type (WT). (PDF) [file pgen.1010945.s023.pdf]

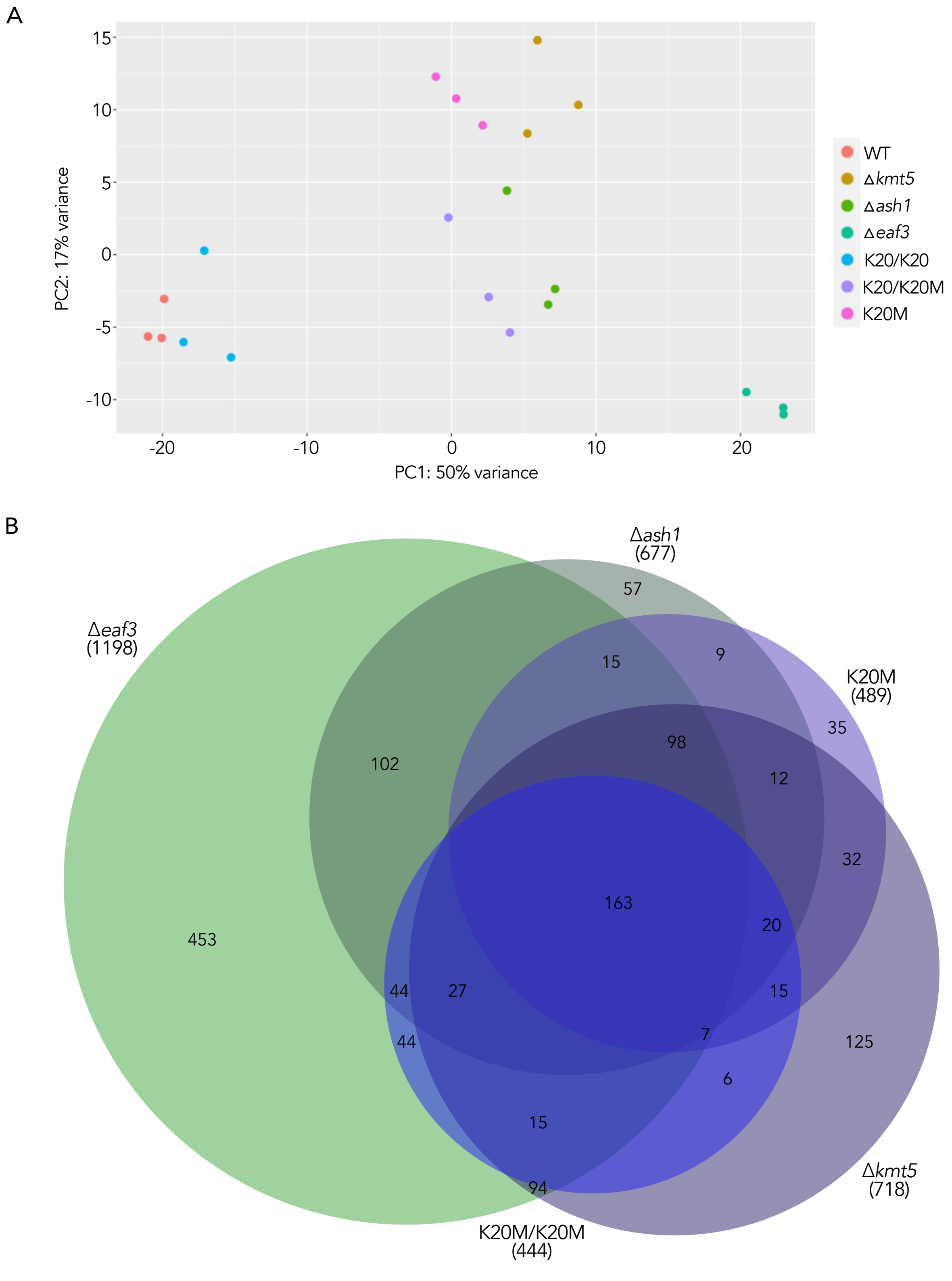

S12 Fig. RNA sequencing of mutant strains. A) Principal Component Analysis (PCA) plot of all sequenced replicate strains. B) Venn diagram of upregulated genes (DESeq2, padj ≤0.01, | log2 fold change | ≥0.585) in all mutants compared to wild type (WT).
