# Supplementary material for: H4K20me3 is important for Ash1-mediated H3K36me3 and transcriptional silencing in facultative heterochromatin in a fungal pathogen
Source: PLoS Genet. 2023 Sep 25;19(9):e1010945. doi: 10.1371/journal.pgen.1010945 (PMC10553808; doi:10.1371/journal.pgen.1010945)
Supplement: S13 Fig — Pairwise sequence similarity is noted below the alignments and ranges from 55 to 78%. Light grey represents agreements to the reference and black represents disagreements. (PDF) [file pgen.1010945.s024.pdf]

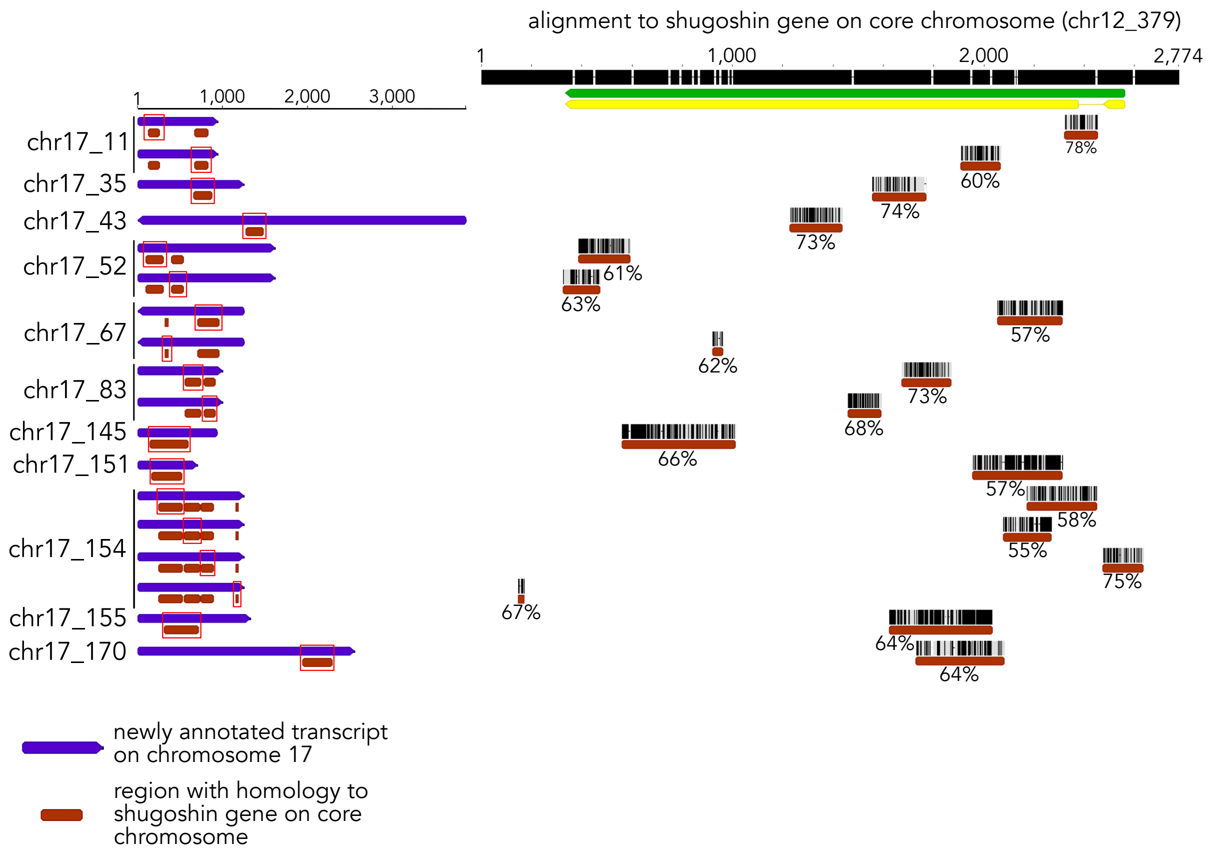

S13 Fig. Alignment of sequences showing similarity (red) to the gene encoding Shugoshin on core chromosome 12 that were identified in newly annotated transcripts (purple) from chromosome 17 to the *shugoshin* gene sequence. Pairwise sequence similarity is noted below the alignments and ranges from 55 to 78%. Light grey represents agreements to the reference and black represents disagreements.
