# Supplementary material for: H4K20me3 is important for Ash1-mediated H3K36me3 and transcriptional silencing in facultative heterochromatin in a fungal pathogen
Source: PLoS Genet. 2023 Sep 25;19(9):e1010945. doi: 10.1371/journal.pgen.1010945 (PMC10553808; doi:10.1371/journal.pgen.1010945)
Supplement: Attachment — Submitted filename: response_reviewers_FINAL.pdf [file pgen.1010945.s025.pdf]

Reviewer #1: Review of "H4K20me3 controls Ash1-mediated H3K36me3 and transcriptional silencing in facultative heterochromatin"
Möller et al.
PLoS Genetics
050823

In the manuscript "H4K20me3 controls Ash1-mediated H3K36me3 and transcriptional silencing in facultative heterochromatin", Möller et al. characterize the histone post-translational modification (PTM) H4K20me3 as being important for the establishment and transcriptional repression of facultative heterochromatin in the filamentous fungal organism Zymoseptoria tritici, a critical pathogen of wheat. The authors show that the deposition of H4K20me3 by the enzyme KMT5 controls the deposition of H3K36me3 by ASH1 and can influence the enrichment of H3K27me3. Together, these data argue that the action of KMT5 to trimethylated H4K20 is epistatic to ASH1 deposition on H3K36 and can influence the activity of the PRC2 complex for trimethylation of H3K27; these conclusions were reached using both strains containing full deletions of the histone methyltransferases and in strains encoding histones with the requisite lysine mutated (either as the only copy or as ectopic copies in tandem with the endogenous histone H4 gene). The loss of H4K20me3 and H3K36me3 in Δkmt5 strains and movement of H3K27me3 then influence the expression of genes in formerly facultative heterochromatic regions, with hundreds of non-coding genes (based on the presence of stop codons in their reading frames) that had homology to protein-encoding genes on the core chromosomes being upregulated. These novel transcripts argue that Z. tritici employs transcriptional gene silencing with these non-coding transcripts to modulate gene expression, possibly in different developmental stages or in response to different environmental conditions - a fascinating mechanism that should be explored further in later manuscripts.

Overall, I felt this manuscript presented a clear story of the histone PTMs ("epigenetics") of Z. tritici and the epistatic nature of the H4K20me3 mark controlling ASH1-specific H3K36me3 and influencing H3K27me2/3 deposition and gene expression. In general, the authors performed multiple ChIP-seq and RNA-seq experiments that were convincing of the role of these enzymes in establishing the repressive facultative heterochromatin. The authors also added in Hi-C data in the supplemental data, and given the paucity of information about it, it seems appropriate to have these data in the supplement (although I have questions about it). The genomics experiments are sufficient for this manuscript, and while I think some biochemical experiments could enhance the story, I agree with the authors that they are better suited for a later manuscript. Overall, I think PLoS Genetics is the appropriate journal for this manuscript. However, I feel there are some places in this manuscript where the authors could have elaborated with additional clarifying information. Also, another figure that specifically shows the potential interactions between their novel non-coding RNAs and the corresponding protein-encoding genes in the core chromosomes would go a long way towards explaining this observation better. Specifically, more information is needed about the non-coding RNAs in facultative heterochromatin, including directly stating that these are polyadenylated, given your methods to isolate these non-coding RNAs use oligo dT. Why are they polyadenylated

(perhaps the authors could speculate in the discussion)? Would these non-coding RNAs be thought to act through basepairing interactions with their targets? Would they be expected to be processed into small interfering RNAs by Dicer or Argonaut (or, even more basic, are there homologs for Dicer and Argonaut encoded in the Z. tritici genome)? This specific section must be elaborated with additional text/information so that it is abundantly clear to the reader how these non-coding RNAs were discovered (without having to dig deep in the supplement to find out that they are polyadenylated) and what these non-coding RNAs could be doing. Some information that details the action of those non-coding RNAs would be beneficial to discuss beyond the one sentence that was currently written in the Discussion ([lines 472-474]: "How exactly these RNA species act, what their specific functions may be, and whether they interfere with or aid propagation of accessory chromosomes in Z. tritici are interesting questions but go beyond the scope of the current study.").
I did have some major and minor concerns regarding this manuscript, which if addressed, should not impede publication:

Major concerns:
1. As detailed above, my biggest concern came with the paucity of information regarding the non-coding RNAs encoded on the accessory chromosomes in Z. tritici. The paragraph (lines 320-348) describing the identification of these non-coding RNAs really piqued my interest, but there is no evidence associated with it. I think that some (two?) alignments of non-coding RNAs (with stop codons boxed) and their possible protein-encoding binding partner on the core chromosome could be shown to elaborate on these observations (perhaps that is what Figure 6C is for; this figure is not referred to in the manuscript at all). The Results section text should also specifically state that these non-coding transcripts must be polyadenylated given the protocols used for isolating and sequencing these RNA species, as well as how they were discovered: if these transcripts are homologous to genes on the core chromosome, how did they map to the accessory chromosome? I am assuming there was enough sequence divergence to allow mapping to two different sites in the Z. tritici genome? This needs to be fully explained (again, perhaps Figure 6C is meant to elaborate this point, but if so, the same read mapping data should be shown for the core chromosomes to highlight the unique reads present at those two loci - and zoom in so that two or three genes are shown). And as noted above, some speculation about the mechanism of processing and targeting for these non-coding, regulatory transcripts would be beneficial to the reader. As presented, this section is insufficient for an understanding of this observation.

We would like to thank Reviewer 1 for the generally favorable and in-depth review of our manuscript, and we are happy to see that they think of PLOS Genetics as the appropriate journal for this work. We, of course, share the reviewer's interest in transcripts emanating from the accessory chromosomes when Kmt5 is lacking (they were discovered as novel and unexpected transcript by RNA-seq in the *kmt5* mutant). As other reviewers have noted, inferring possible functions of these RNAs from the available data is still speculative, so we toned down and shortened this section while also providing more clarifying information as

requested by Reviewer 1. We'd also like to remind Reviewer 1 that we do not actually know yet whether these RNAs are "non-coding" or not (even though we suggested this in the original abstract, from which we removed the word "non-coding"). In fact, much of what Reviewer 1 proposes in the "major concerns" would be excellent ideas for an entirely new project, where one of the first aims would be to ascertain whether these novel RNAs generate short peptides, for example. However, any of this work far exceeds the scope and purpose of the current manuscript.

Based on comments by other reviewers, we shortened these sections but we now state that these RNAs are very likely mostly polyadenylated (main text, Line 301, 332 and elsewhere), even though non poly(A) RNA is always obtained as background, just like rRNA, with the method used. We also generated an additional supplemental figure showing examples of novel, truncated transcripts with sequence similarity to a core chromosome gene (S13 Fig); the figure shows typical sequence divergence between the sequences on accessory and core chromosomes, and we found that incorrect mapping is not a concern, especially since we used paired-end 150-nt sequencing.

There are both Dicer and Argonaute homologs in *Z. tritici* (see Habig et al., 2021: "Ago1 Affects the Virulence of the Fungal Plant Pathogen Zymoseptoria tritici"). With the limited data we have no clear indication of the putative (if any) function(s) of these RNAs, and other than reporting that they are produced in *kmt5* mutants we prefer not to speculate. It was unclear to us whether Reviewer 1 wished to see a biochemical (i.e., binding of specific RNAs to the proteins) or a genetic study (i.e., presence of the novel RNA in either *dcl* or *ago* mutants), but either or both of these projects are well beyond the scope of the current work. *Zymoseptoria* biochemistry is poorly developed, and we cannot cross mutants in the laboratory yet, so that time-consuming and cumbersome transformation experiments would need to be done to address these issues.

The fact that we find multiple, fragmented copies of these putative genes on several accessory chromosomes, and the finding that only short regions of the transcripts produced show sequence similarity to core chromosome genes may indicate that they represent ancestral duplications of core genes on accessory chromosomes that were mutated and fragmented due to frequent rearrangements. While these regions can be expressed when H4K20 methylation is absent, we do not yet know whether absence of H4K20 methylation alone allows for transcription or whether other "signals" are needed. In summary, the major point of the section on RNAs that are produced in absence of Kmt5 was to report the increase of transcription in general, and to at least provide an initial characterization of these novel short transcripts that had not been previously shown to exist.

2. As alluded to above, the Hi-C data does not have enough information for interpretation. As presented, you have an observed vs. expected contact map, which shows some facultative heterochromatic regions having less interactions with surrounding chromatin. However, additional information is needed for readers to interpret these data, including the number of

valid reads produced from the experiment that went into the contact matrix (not just the toal number of reads) and the resolution of the images (what are the bin sizes?), as well as images of the contacts across the genome in raw and Knight-Ruiz corrected datasets (are the matrices used for the observed-expected heatmap presented in the supplemental information Knight-Ruiz corrected? This is critical to eliminate biases in the contact heatmap that might be due to underlying sequence differences or differences in DpnII site locations), which would be critical to understand if the Z. tritici genome also employs a Rabl chromosome conformation like other fungal systems (e.g., S. pombe, S. cerevisiae, N. crassa, V. dahliae, etc.); the heatmaps shown here look like only a couple of chromosomes have contacting centromeres, which would be unique among other high-quality fungal Hi-C datasets where all centromere contacts are uniform between all chromosomes. The authors also need to state in their manuscript, preferably in the results section, that the Hi-C data was collected using proximity ligation, which is known to be less accurate than in situ Hi-C in which ligation products are captured in the nucleus and as such are more apt to reflect valid contacts. This information would be helpful to clarify the genome organization of Z. tritici, which to my knowledge, is not known. Lastly, lines 173-176, in the authors state that the facultative het region interactions persist in the absence of H3K27me3, but from what I can tell, the Hi-C data presented are just of a WT strain. How can the authors conclude that H3K27me3 plays no role? Am I missing a manuscript in which this is referred (if so, please add that information)? Please revise this entire section to more accurately detail the Hi-C data, and/or to explicitly detail the Δkmt6 Hi-C data if it is presented here.

Based on the suggestions of several reviewers (see below), we removed the Hi-C data from this manuscript.

3. Do the authors have any idea why H3K27me3 is not ablated in a Δkmt5 mutant? Certainly, with the loss of H4K20me3, some regions lose H3K27me3 and others have a slight redistribution of that mark. These data argue that there is some (probably indirect) control of H3K27me3 by KMT5, but it is unclear to what extent. Could the authors speculate in the discussion more about what the redistribution of H3K27me3 could mean? Is it possible that the PRC2 complex binds H4K20me3 for normal localization? Is there information about whether PRC2 interacts with Eaf3 or Ash1, possibly from other species? At the very least, there should be a greater discussion of the results in Bicocca et al., which detail how some facultative heterochromatic regions in N. crassa have co-localization of both ASH-1 specific H3K36me2 and H3K27me2/3; it is possible that the facultative heterochromatin regions most affected in a Δkmt5 strain could be those that present with overlapping H3K36me3 and H3K27me2/3. Perhaps the authors could take a bed file with H3K36me3 domains and ask whether the H3K27me3 enrichment is strongly changed in these regions in a Δkmt5 strain relative to a WT strain? In general, the changes of H3K27me3 in a Δkmt5 strain should be addressed a more in this manuscript.

We are not aware of any studies that show a direct connection between H4K20me3 and H3K27me3 (or the PRC2 complex). The changes in H3K27me3 that we observed may be due

to chromatin remodeling upon transcriptional activation in facultative heterochromatin, indirect effects of differentially expressed genes in the mutant or change of chromatin dynamics in absence of all H4K20me. Since our study does not address the impacts of the other methylation states of H4K20 or the role of H4K20 methylation outside of facultative heterochromatin, there are many possible factors that may play a role here. We do show that Ash1-specific H3K36me3 and H3K27me3 overlap (these are the regions we define as "facultative heterochromatin") and we now provide additional analyses including peak calling and defining domains of Ash1-mediated and Set2-mediated H3K36me3 and mention the *N. crassa* ASH-1 paper in the discussion. However, H3K27me3 dynamics in fungi are not well understood and even with our data it is still difficult to disentangle the specific changes in H3K27me3, as they seem to differ even within the genome. We are not keen on "speculat(ing) in the discussion".

Minor concerns:
1. Figure 6C is not referred to in the results text. Please add that text, in addition to the suggestions I have provided above.

Thanks for noticing this! Figure 6C is now referred to in the text (line 334) and we added an additional supplemental figure (S13) addressing sequence similarity between accessory chromosome transcripts and core chromosome genes.

2. I think the manuscript would be better off with a model of possible action. You have one in Figure S11; I don't think this should be buried in the supplemental material. I would suggest moving that figure to the main figures. Doing so will help the reader understand the epistatic depositions of histone PTMs in facultative heterochromatin.

We moved the model figure to the main text (Fig 7), also based on a suggestion by a different reviewer.

3. Line 41: "…to understand epistasis relationships…" epistatic?

We would prefer to leave this sentence as it is, because we think "epistasis" is correct in this context.

4. Line 46ff: this run-on sentence is really long and quite difficult to understand. Please break it up.

Done!

5. Line 233ff: is there any information on what these regions could be doing to still have H4K20me3 in the cells with two copies of the histone H4 genes? Are there specific signals? Correlated to epigenetic marks or Hi-C contacts? Perhaps add any information you have to the discussion.

Unfortunately, we do not have any additional information on specific signals in these regions besides what is already stated in the manuscript. They are regions we defined as facultative heterochromatin, so usually enriched with H3K27me3, H4K20me3 and Ash1-mediated H3K36me3, and seem to be preferentially methylated in some histone mutant strains and the tagged *kmt5* strains. It is possible that these regions indicate domains that are methylated "early" but we have not shown this yet.

6. Line 235: the authors state that the "…presence of H4K20me3 appears to be necessary to deposit H3K36me3 and in some regions H3K27me3". But there are still extensive regions of the genome with enrichment of the downstream regions. As stated, this seems like an "all or none" effect, but it's more complex than that. Please clarify and/or revise this statement.

Changed to "is important", but we also state that this only applies to some regions, so it is not an "all or none" effect.

We also have changed the title as suggested (by Reviewers 2 and 3), and use "important" rather than "essential" to characterize the impact of H4K20me3 on H3K36me3.

7. Line 243ff: please clarify with fusion (N or C or both?) of KMT5 produces that observation noted in this sentence.

Thanks for noticing this; we added that information (line 267).

8. Line 294ff: the authors denote the number of genes with changes in gene expression in a Δkmt5 strain relative to a WT strain. Are all these genes found in facultative het regions, or are they enriched with H4K20me3, H3K36me3, or H3K27me3? It is unclear if these numbers are the total changed genes or just those in facultative het regions (as written, I would believe the former). It would be great for the authors to clarify how many genes may have direct changes in expression, specifically reporting the genes that are enriched with any (or all) of the facultative heterochromatin marks and showing an example or two of genes that could be subject to direct changes in facultative heterochromatin.

We list both, all differentially expressed genes (shown in Fig 6A) and specifically genes in facultative heterochromatin. We have clarified this in the figure legend. Based on our findings that most currently available gene annotations are incorrect in facultative heterochromatin regions, it is not straightforward to quantify direct and indirect changes, but we addressed this by listing the number of genes that are differentially expressed in facultative heterochromatin (which we defined as enriched with H4K20me3, H3K27me3 and H3K36me3 in wild type) and their relative frequency compared to all differentially expressed genes (see this paragraph (line 313ff: "Out of 718 upregulated genes (DESeq2, padj ≤0.01, | log2 fold change | ≥0.585, Fig 6A) in Δ*kmt5*, 286 were located in facultative heterochromatin (~40% of upregulated genes). Similarly, 39% of upregulated genes in the K20M mutant were within facultative

heterochromatin, indicating that Kmt5 and H4K20me3 are especially important for gene repression in those regions. While all mutant strains we included showed significant enrichment of upregulated genes in facultative heterochromatin (chi-square ≥0.001 for all mutants), the relative proportion differs (26% in Δ*ash1*, 19% in K20/K20M, and 15% in Δ*eaf3*).” However, there are also many differentially expressed genes outside of facultative heterochromatin regions.

9. Line 425ff: as written, this statement suggests that Eaf3 somehow represses KMT5 activity. Is this what the authors are concluding (e.g., without Eaf3, KMT5 is capable of continued trimethylation)? Please clarify.

Yes, this is one possible scenario. We clarified this by adding: “This is supported by finding increased H4K20me3 levels in the absence of Eaf3, which may be a result of continued methylation of H4K20 by Kmt5 in the absence of Eaf3 binding to H4K20me3 and the requirement of Eaf3 for Ash1-mediated H3K36me3” (Line 440ff).

10. Line 497: please subscript the “2” in MgCl2.

Thanks for finding this typo. Done!

11. The reviewer links for the NIH Bioproject do not work (they seem to be duplicate links), but I can access the zenodo data. What I really wanted to see was whether the authors provided the .bed files for the genome features they evaluate here, as well as the contact matrices for your Hi-C data, which are not present in the zenodo database (but may be in the SRA [the GEO might be better?], perhaps as supplemental files somehow?). Please make sure you provide not just the sequencing reads, but the Hi-C contact matrices and the bed files for enriched regions to your readers.

We are sorry the links were not working. We have updated the links and the data should be publicly available soon. Currently, the SRA reviewer links allow to view all metadata of deposited datasets. We have also added ChIP-seq tracks for the complementation experiments, bed files of specific regions and peaks to the zenodo submission.

12. In figure 4A, the authors have ChIP-seq tracks with different y-axis scales. Perhaps it would be best to set all scales to the same range for accuracy – this would be important to show how the K20L mutation might seem to cause increased enrichment of H3K20me3, possibly suggesting a stronger interaction of KMT5 with these regions (maybe these data speak to the binding specificity of KMT5?)? Lastly, please be sure to explicitly detail, perhaps in the Materials & Methods, how your ChIP-seq data was normalized (by RPGC, per your supplemental data), so readers know what normalization method was applied.

We tried different scales, but either the enrichment for some tracks becomes difficult to see or others are cut off, so we decided to use different scales. We added a sentence in the figure

legend to emphasize the different scales (line 920). We also added the method for normalization in the methods section and figure legend (line 633 and 919).

Reviewer #2: The manuscript 'H4K20me3 controls Ash1-mediated H3K36me3 and transcriptional silencing in facultative heterochromatin' by Möller and colleagues provides evidence that the histone modifications H4K20me, catalyzed by Kmt5, and H3K36me3, catalyzed by Ash1, are important for facultative heterochromatin in the fungal plant pathogen Zymoseptoria tritici. The manuscript reports on a combination of genomic, genetic, and biochemical experiments to show that the deletion of kmt5 leads to de-repression of expression, which was accompanied by loss of H3K36me3, suggesting that H4K20me3 is important for Ash1-mediated H3K36me3. Loss of H3K36me3 and H4K20me3 led to chromatin reorganization at facultative heterochromatic regions, defined by H3K27me3, H4K20me3 and H3K36me3 in the wild-type strain, thereby providing a mechanism for formation and maintaining of heterochromatin in filamentous fungi.

While the roles of H3K27me3 H3K36me3 in several fungi has been reasonably-well established, the function of H4K20me3 has thus far received far less attention, and the data presented here will be of interest for researchers working on fungal chromatin biology. This submission is generally well-written, the data seems to be robust, and the applied approaches are generally well-suited to support the manuscript's results and conclusions. I would also like to applaud the authors to not only provide the sequencing data but also the scripts as part of the submission.

We would like to thank Reviewer 2 for the in-depth, and largely favorable review of our manuscript.

I, however, also have few essential comments that necessitates the authors' attention:

Loss of Kmt5 or Ash1 lead to several measured phenotypes such as loss of specific histone modifications, changes in chromosomal stability or gene expression changes. Throughout the manuscript, no Kmt5 or Ash1 complementation strains were systematically tested for their capacity to restore wild-type phenotypes. It is mentioned that complementation with tagged Kmt5 does not restore wild-type levels of H4K20me3 (L238fF), which could either indicate that the tags interfere with the correct function or that additional, ectopic effects in occur in the mutants independent of Kmt5. To address this possibility, and I consider complementation experiments essential.

We have now included ChIP-seq experiments and phenotyping with both *kmt5* and *ash1* complementation strains. For kmt5, we complemented with a wild type *kmt5* gene (coming from a different *Z. tritici* isolate), which restores near wildtype enrichment of H4K20me3 and H3K36me3 (S5 Fig). This supports our conclusion that the tagged *kmt5* protein is not fully functional and the tags interfere with protein function. For *ash1* complementation, we integrated a tagged *ash1* gene (*ash1-gfp-V5*) into the *ash1* deletion strain, which restored H3K36me3 levels comparable to those observed in wild type. We added a paragraph in the

results section (line 130ff), a new supplementary figure showing ChIP-seq and phenotyping results (S5 Fig), and uploaded the additional ChIP-seq data to SRA.

Text added (line 130ff): "To complement Δkmt5, we replaced the hph gene with a wild-type kmt5 gene (amplified from isolate Zt469 (34)) and neo resistance gene, conferring resistance to G418. To complement the Δash1 strain, we replaced the hph resistance gene with an ash1-gfp-V5 construct and neo resistance gene. To verify complementation strains restored histone methyltransferase activity, we performed ChIP-seq experiments and phenotyping assays. Complementation strains showed wild-type like H4K20me3 and H3K36me3 enrichment and wild-type like growth under the tested conditions (S5 Fig)."

Based on the description in the manuscript, it seems that the ChIP-seq analyzes were not performed with controls. Since it is not entirely clear if the data has been corrected by e.g., input or no-antibody control, some of the visual enrichments for specific modifications might also be due to background effects. Related to this, I am a bit puzzled why H4K20me3 displays clear enrichment in specific regions, while H4K20me1 does not seem to be enriched (L145). Is it possible that H4K20me1 is largely absent, and that the genome-wide data shows unspecific background? Figure 1 obviously shows the presence of H4K20me1 but are the antibodies specific enough to distinguish H4K20me1 and me3?

We agree with the reviewer that the H4K20me1 enrichment, as assayed by ChIP-seq, does not show strong specific enrichment. We now repeated the H4K20me1 ChIP-seq and also included the Δ*kmt5* strain and an antibody against H4K20me2. We do not see a clear "enrichment in WT" and "absence in Δ*kmt5* pattern" as we do for H4K20me3 for both of these marks. None of the antibodies we have tested so far are validated for ChIP; our lab has more than 15 years of experience running ChIP-seq in close to a dozen systems and our general conclusion is that these antibodies do not work well-if at all-for ChIP in *Zymoseptoria*, one reason why we do not comment much on H4K20me1/me2. We see at best minor reductions in enrichment for both H4K20me1 and H4K20me2 in regions where we detect H4K20me3, which would argue against cross-reactivity between those marks, but with the present data we do not feel confident to assess any specific genome-wide enrichment for H4K20me1/me2. We thus removed the H4K20me1 track from Figure 2.

The manuscript compares protein sequences between different species and inferences potentially diverged functions based on the absence of conserved protein domains or motifs (e.g., L114). Simple sequence similarity searches can easily miss domains/motifs and structural comparisons (e.g., using structure predictions from Alphafold) are much more sensitive to identify diverged yet structurally conserved motifs/domains. To corroborate that absence of domains is linked to divergent functions, structural comparisons of the enzymes studied here would be essential.

We agree that presence or absence of specific motifs by itself does not prove presence or absence of function, and we did not wish to imply this. By various modeling programs, we had

indeed previously looked at the predicted structures of *Zymoseptoria*, *Fusarium*, and *Neurospora* Ash1 and Kmt5 (whole proteins and selected regions of proteins) and -as with so many similar proteins- the only regions that had predicted structure were the SET domains, and the neighboring pre- and post-SET regions. The regions that lacked the motifs mentioned in Figure 1 did not have recognizable structure (i.e., show up as disordered "spaghetti" in Alphafold). We removed the last sentence of the paragraph (*"Absence of these domains in fungal Ash1 homologs suggests different mechanisms of interaction with chromatin."*), now simply stating presence and absence of motifs but no longer speculating about function.

The manuscript concludes 'A homolog of Z. tritici Pdp1 was found to interact with a chromatin remodeling factor, ISWI, in N. crassa, where it also increased H3K27me3 levels; thus Z. tritici Pdp1 is a putative homolog of S. cerevisiae Ioc4.' Based on the information provided, it is not clear how this conclusion is derived? Is loc4 a homolog/ortholog of Pdp1 in Neurospora/Z. tritici? It seems that the results and discussion here focus on functional equivalence rather than evolutionary relatedness, the latter which is key to determine if sequences are homologs/orthologs. Thus, what is a 'true ortholog' or 'best homolog' (L432) according to the authors? To disentangle this in the results and discussion, the manuscript needs to show an alignment of PWWP domain containing proteins (or just the domains) and a phylogenetic analysis to determine which sequences are orthologs, and then superimpose the potential function; proteins with distinct functions can be orthologs and homologs with similar function might still be paralogs.

Based on our sequence analyses, "Pdp1" is the only PWWP domain containing protein in the filamentous fungi we looked at. In *Neurospora crassa*, this protein, originally called "CRF4-3" but now referred to as "IAF-3" (Wiles et al, 2022 eLife; Kamei et al, 2021, PNAS) was found to interact with ISWI, like budding yeast Ioc4 does. Based on the observation that there is only one PWWP domain containing protein, we suggest that the protein found in *Z. tritici* (as in other filamentous fungi) is most likely an ortholog of *N. crassa* IAF-3, which is by sequence and function related to budding yeast Ioc4. Because we found that *Zymoseptoria* "Pdp1" had no effect on H4K20 methylation we decided not to add a figure that shows alignments. The reviewer is correct, of course, sequence similarity does not mean that a protein carries out similar functions. We changed the text accordingly.
Changed text (line 275ff): *"Deletion of the respective gene in Z. tritici did not affect H4K20me3 or H3K36me3 (Fig 5B and C) but rather led to increased enrichment of H3K27me3 in numerous regions along the genome (S7, S8, S11 Fig), suggesting that there is no PWWP domain-containing ortholog of the S. pombe Pdp1 in Zymoseptoria. A homolog of the Z. tritici Pdp1 (PWWP domain-containing protein 1) was found to interact with a chromatin remodeling factor, ISWI, in N. crassa, where it also increased H3K27me3 levels (40). Based on the similarity among the Zymoseptoria, Neurospora, and Fusarium Pdp1-like proteins, we propose that these proteins are orthologs of the Saccharomyces cerevisiae protein Ioc4 (40)."*

The importance of the co-localization of heterochromatic regions to the presented story is not clear. Co-localization of H3K27me3-rich regions have been previously reported for some fungi

(e.g., Neurospora crassa or Verticillium dahliae), and thus the observation that regions enriched for H3K27me3, and with H3K36me3 and H4K20me3 as a consequence of the overlaps, are interacting is not surprising per se, as is the notion that interactions are maintained in the absence of H3K27me3 (has been shown previously for N. crassa). However, Kmt5 and/or Ash1 mutants were not assayed with Hi-C, and thus their role in maintaining the 3D chromatin organization remain unclear. These experiments would be essential for the presented narrative, and in the absence of these, I would suggest removing these data from the current submission.

As suggested by the reviewer, we removed the Hi-C data from the manuscript.

Lastly, I would suggest adjusting the manuscript's title such that it clarifies that the manuscripts focused on filamentous fungi.

We changed the title of the manuscript, according to the suggestions of Reviewers 2 and 3 to "H4K20me3 is important for Ash1-mediated H3K36me3 and transcriptional silencing in facultative heterochromatin in a fungal pathogen".

Additional detailed comments and suggestions aimed to further improve the manuscript (in no specific order):

L19: 'Reversal of silencing…' would it maybe be clearer to say 'De-repression…' ?

Changed to derepression.

L49: '…specific genes…' What is meant here by specific genes? Could the manuscript please be more precise what defined these set of genes?

We added that these are often -but not always- genes involved in development (line 52).

L76-82: it should be clarified that most fungi, if not all, have SET2, but that only a subset seem to also have ASH-1 (see e.g., Bicocca et al., 2018, Elife; Zhang et al., 2022, Microb. Genomics). The emphasis here is on ASH-1, which makes sense given the data presented, but the current narrative might suggest that species might only have ASH-1, which should be clarified. Moreover, the importance and function of SET2 and the interaction between H3K4me and SET2-catalyzed H3K36me should be explained more extensively. Lastly, the distinct functions of SET2 and ASH-1 should be mentioned (see e.g., Ren, et al., 2021, Journal of Fungi; Ferraro, et al., 2021, BMC Genomics).

We have clarified that Ash1 is, in some organisms, found in addition to Set2 in the introduction (line 79ff). We also had previously included some of the suggested references but now added additional information about Set2 in the results section, and we now differentiate between Ash-1- and Set-2-mediated H3K36me3. However, we did not add more extensive details about the interaction of H3K4me2 and Set2-catalyzed H3K36me3 as that is not the focus of this study

and was not addressed by experiments.

L109: the manuscript cannot claim that Kmt5 is reasonable for all H4K20 methylation (Figure 1) as H4K20me2 was not assessed. To support this statement, additional ChIP-seq experiments and/or (at least) Western blots (as in Figure 1B) are required; alternatively, statements concerning the breadth of activity of Kmt5 need to be toned down.

While the anti-H4K20me1 antibody does not work (well) for ChIP, it does work for western blots. Based on our western blot data, which show absence of H4K20me1 and H4K20me3, reports in *Fusarium* spp. (Bachleitner et al., 2021, Fungal Genetics and Biology) that Kmt5 is responsible for all H4K20me, and the lack of any other known or potential genes for Kmt5 homologues, we argue that it is extremely unlikely that there is another H4K20 HMT in *Z. tritici*. However, since our H4K20me1/2 ChIP-seq data are inconclusive, and we have not found a good antibody against H4K20me2, we toned down the statement from "Kmt5 is responsible for all H4K20 methylation" to "Kmt5 in Z. tritici is most likely responsible for all H4K20 trimethylation" (line 114ff).

L128ff: Loss of Kmt5 and Ash1 increases chromosome loss compared with the wild-type situations (Table 1). Can the authors speculate why specific chromosomes display increased loss rates? What are their features that distinguishes them from other accessory chromosomes.

We have reported on accessory chromosome loss in other HMT mutants before (Möller et al., 2019), where we tried to find characteristics that differentiate accessory chromosomes that are frequently lost and infrequently lost. Unfortunately, we have so far been unable to identify distinct features. We have added this statement to the chromosome loss section: "We have not found any specific characteristics that make some accessory chromosomes more prone to be lost in certain mutant backgrounds." (line 144ff).

L140: it would increase readability if the manuscript would directly explain which histone modification were assayed using ChIP-seq.

We now listed the tested modifications at the start of the paragraph (line 150).

L158: The co-occurrence of H3K9me3 and H3K27me3 at specific loci has been previous reported by Schotanus and colleagues (2015), which should be also mentioned here.

We added the suggested reference to the paragraph (line 173).

L157: The composition of cluster 1 and 2, also in respect to the precise numbers/fractions of other histone modifications such as H3K9me3 need to be reported.

Clusters 1 and 2 are based on enrichment or lack of H4K20me3 and H3K36me3 in H3K27me3 enriched regions. The composition of clusters in terms of gene and repeat content is shown in

Fig 2C. As cluster 2 contains quite a few TEs, there is also considerable enrichment with H3K9me3, as mentioned in the text. As shown by ChIP-seq, there is overall very little H3K4me2 in regions with H3K27me3 or H3K9me3. We do not see why adding "precise" fractions of other histone modifications would benefit the analysis.

L190: the manuscript indicates that '…at least one H3K36 methyltransferase is essential.' Based on the phenotypes of the mutants, it seems that indeed SET2 and not ASH-1 is essential, which is in line with previous work (see above), and should be clarified.

Since we did obtain Δ*set2* mutants, we cannot consider Set2 essential, even though the mutants grow extremely poorly. We were, however, not able to generate Δ*ash1*/Δ*set2* double mutants, which suggests that it is indeed "required" for *Z. tritici* to have at least one Set2-type HMT present. Changed text: *"We obtained single Δset2 mutants that showed severe growth defects when compared to wild type (S9 Fig), but we were unable to obtain mutants for this methyltransferase in the Δash1 background, suggesting that the presence of at least one H3K36 methyltransferase is required in Z. tritici."* (line 19ff).

L217: why was lysine 20 replaced by these specific amino acids. Could the manuscript please provide some insights?

We chose methionine because other studies suggested this works as a methyltransferase inhibitor, glutamine as a mimic of an acetylated lysine and leucine as a slightly smaller, non-modifiable amino acid.
Changed text: *"…we constructed histone H4K20 mutants, replacing lysine residue 20 with either leucine (L, as a non-modifiable, relatively large residue), methionine (M, as a potential inhibitor of methyltransferase activity) or glutamine (Q, to mimic an acetylated lysine)."* (line 230ff).

L293: it is not clear if the 718 reported genes are differentially regulated or indeed up regulated in the mutant. The text says the former, the notion that the log2FC > l 0.585 l suggests that latter. Please clarify. Where is the cutoff (0.585) coming from?

The 718 genes refer to all differentially expressed genes. We clarified the parameters by adding this: "(DESeq2, padj ≤0.01, l log2 fold change l ≥0.585)" to the text (line 313). We chose 0.585 to filter for 1.5x fold change.

L323: Why focus on chromosome 17 for manual annotation and not for the other accessory chromosomes? Would the picture look any different if more or different accessory chromosomes would be analyzed? What is the function of the anti-sense transcripts if no correlation with gene expression levels of homologs genes could be observed? This section necessitates either a more systematic analyzes or should be significantly shortened.

We focused on chromosome 17 because it is present in high frequency in *Z. tritici* populations (in contrast to some other accessory chromosomes that exhibit a much higher presence/absence variation), shows strong enrichment of facultative heterochromatin marks (H3K27me3, H3K36me3 and H4K20me3) and has a moderate TE content. We would expect some variation in number of this type of transcripts found on different accessory chromosomes, as some are more TE-rich (chromosome 14) and some have a higher proportion of euchromatin (chromosomes 20 and 21). However, we did observe these types of transcripts arising from all accessory chromosomes and facultative heterochromatin regions on core chromosomes, so we would consider them a common feature of these regions.

To address the functional part, as detailed in the response to reviewer one, we shortened the section about possible functions, as we cannot assign any functions with the available data, and added an additional supplemental figure (S13 Fig) to visualize sequence similarity between those transcripts and core chromosome genes.

L416: I would suggest, if possible, to move the model in Figure S11 to a main figure to further support the clarity of the discussion.

As also suggested by Reviewer 1, we moved the model to the main text (Fig 7).

Figure 1: Does Neurospora crassa lack KMT5? Otherwise, it would be useful to add the cartoon of the protein here as well. Moreover, it might be useful to swap panel B and C to fit the overall narrative of the manuscript.

Neurospora crassa does have a Kmt5 homolog, but since there are no studies on Kmt5 in *N. crassa*, we did not include it here because it would not add additional information. We feel that the current order is fine because we first talk about Kmt5 homologs.

Figure 2: I would suggest changing the order in panel to H3K27me3, H3K27me2, H4K20me3, H4K20me1, H3K36me3, H3K9me3, and H3K4me2 to better fit the narrative in the manuscript.

We decided to leave the order of shown ChIP-seq tracks as is (but removed the H4K20me1 track), as this is in line with the order of addressing them in the text and with the other figures in the manuscript.

Figure 3: Transposable elements should be shown, analogous to the display in Figure 2. It is not clear how the fragments in the heatmap in B was ordered; I assume from high to low for the WT, yet this is not fully explained (this also applies to the other figures).

Transposable elements are now added for this figure as well. Yes, the fragments are ordered from high to low enrichment in WT. We have clarified this in the figure legend.

Figure 4: in panel C and D it is unclear which of the heatmaps display the wild type. Could these please be added (either via labelling or by adding the wild-type data for clarity)?

We have modified the figure and added * to the respective control (wildtype) strains in this experiment and clarified this in the legend. We reasoned that having these strains as controls would be better in this experiment to test if integration of an additional *hH4* or deletion of the endogenous *hH4* had any obvious effects. Both strains resembled the normal wild type.

Figure 5B: The wild-type heatmaps seems to differ from the one in Figure 3. How is this possible as supposedly the same data is shown?

This is an astute observation! By including more datasets (Δ*eaf3* and Δ*pdp1*) in calculations for this heatmap, the scales change as the Δ*eaf3* mutant shows more H4K20me3 enrichment than the other tested strains. That is why it seems like there is less enrichment in WT.

Figure 6: from panel B it is not entirely clear if all genes are coming from H3K27me3 enriched regions or only a subset of those shown. Could the numbers be added to the legend or figure?

The figure shows newly annotated genes per strain in facultative heterochromatin and the number is given on top of each panel. We have made this clearer in the figure legend.

Reviewer #3: The manuscript by Moller and colleagues reports on genetics, genome-wide distribution, transcriptional impact, and biological aspects of heterochromatin formation in the fungal pathogen Zymoseptoria tritici. The focus of the research is the interplay between H4K20me3 and H3K36me3 deposited by Kmt5 and Ash1 respectively. Understanding heterochromatin formation and function is of great importance to genome biology, and of specific interest in fungal biology. There are many questions related to fungal development and evolution, along with environmentally regulated transcription. The results reported here provide new insights into hierarchy and cross-talk between histone modifications contributing to heterochromatin formation. I enjoyed reading the paper, and it makes important contributions to the literature of heterochromatin function.

I read the included reviews from previous submission, which did raise valid concerns. However, I agree with the authors assessment that they are good suggestions for future work. The genetic conclusions regarding 'requirement' of specific proteins for individual histone modifications can stand on its own, provided the conclusions are discussed as such.

My overall comments regarding the manuscript are related to the title and reliance on chromatin maps to make genome-wide claims about requirements, interactions, and functional impacts. The word 'control' in the title is too strong given the previous reviewers concerns, and some contradicting data in the paper. The results and claims would be more interpretable with clearer quantification of chromatin domains, their changes in mutants, and reported impacts.

We would like to thank Reviewer 3 for the favorable review of our manuscript. We have changed the title as suggested (also by Reviewer 2), use "important" rather than "essential" to characterize the impact of H4K20me3 on H3K36me3, and added quantification (see below).

Specific comments are listed below.

1. Lines 96-98: "Kmt5 and H4K20me3 are crucial for transcriptional silencing in regions of facultative heterochromatin and essential for Ash1-mediated H3K36me3, thereby contributing significantly to formation of H3K27me3-enriched facultative heterochromatin." I am not sure why the authors use the term "H3K27me3-enriched facultative heterochromatin". Is the point to link the results directly to H3K27me3, or to try and distinguish this specific type of heterochromatin? The point about different types of heterochromatin is a bit of a stumbling block for me in the paper. It is clear that not all heterochromatin is the same, but are there actually good data in the literature regarding 'facultative' heterochromatin in fungi? I interpret the term to imply dynamics, where the chromatin is switching states, but this lack empirical data in fungi. Or the authors can provide references. Otherwise, it seems important to note that the description of facultative heterochromatin is not actually about labile chromatin or DNA accessibility, but rather the presence of histone marks.

We agree with the reviewer that facultative heterochromatin is not well defined in fungi and the function of this type of chromatin may vary between species. Therefore, we decided to "label" chromatin based on the presence of certain histone modifications rather than any functional enrichment (such as developmental genes in animals) or labile chromatin, as our currently available data do not allow for this type of characterization. We added a brief clarification in the paragraph above this statement that we define facultative heterochromatin in this species by presence of H3K27me3 and transcriptional repression (line 91ff).

2. It is not a major conclusion in the paper, but it is not possible to assess the comments regarding Hi-C and interdomain interactions (Lines 171-175). There needs to be some analysis to support the statement about strong interactions between cluster 1 regions. It might also stand better to leave the impact of HiC for a different paper with more complete analysis on all the types of interactions. Interpretation of this type of figure is too subjective.

Based on the comments of the reviewers, we decided to remove the Hi-C data from the current study.

3. Have the authors considered calling domain peaks, such as with the common and well documented tool MACS2? This is not without its caveats, but the reader needs some quantitative/qualitative assessment of changes between these mutants to assess the genome wide claims made.

We have called peaks now using HOMER (see methods part) to identify and quantify regions in the genome enriched with H3K27me3, H3K36me3 and H4K20me3 in WT, Δ*kmt5* and Δ*ash1*

strains. We have added a supplemental figure (S10 Fig) and table (S1 table) showing the % coverage of each histone mark per chromosome. All changes affect the accessory genome to a much higher extent than the core genome, supporting our conclusions that there is a strong impact on facultative heterochromatin.

Added text: *"We performed peak calling to quantify how much of the genome-wide H3K36me3 is mediated by Ash1 and Set2. Based on comparisons of wild type and Δash1 H3K36me3 peaks, ~10% of H3K36me3 in wild type is not detectable in Δash1, indicating that Set2 is responsible for the vast majority (presumably ~90%) of H3K36me3, which aligns with previous reports in N. crassa (30). However, this number may not fully represent Ash1-mediated H3K36me3, as in the absence of Ash1, Set2 may move to regions that are usually targeted by Ash1 in wild type. While Ash1-mediated H3K36me3 comprises less than 5% of H3K36me3 on core chromosomes (except for chromosome 7), Ash1 seems to be responsible for 50-95% of H3K36me3 on accessory chromosomes (S10 Fig, S1 Table). H4K20me3 overlaps with both, Set2-mediated H3K36me3 and Ash1-mediated H3K36me3, but we found a stronger correlation between Ash1-mediated H3K36me3 (~98% of domains overlap) than Set2-mediated H3K36me3 (~80% of domains overlap)."* (line 196ff).

4. Can the authors provide some assessment of how much H3K36me3 signal is lost in ash1-KO. I appreciate they do not think WB will be informative, but the genome wide ChIP can be used to directly assess this. Either base pairs covered in WT versus ash1, or the number of peaks lost? This would help confirm that Ash1, and presumably Set2, function similar to reports in other fungi in terms of genome coverage and chromatin compartments.

We have included this information in the above mentioned new supplemental figure and table (also see reply to comment #3). Based on this data, H3K36me3 covers ~53 % of the genome. Set2 is (presumably) responsible for ~90%, while Ash1 is responsible for ~10 % of genome wide H3K36me3 with a big difference between core (Ash1 responsible for <5%) and accessory chromosomes (Ash1 responsible for 50-90%). However, in absence of a tractable Δ*set2* it is difficult to fully determine all regions that are Ash1-dependent in wild type since Set2-mediated H3K36me3 may move into these regions in an Δ*ash1* mutant, especially when formerly repressed regions become transcriptionally active.

5. Following from the previous comment, the analysis would benefit from clearly distinguishing H3K36me3 that is Ash1-dependent versus not dependent, and how this relates to chromatin and H4K20me3. For instance, in Fig. 3A, the left panel shows that the majority of H3K36me3 in the region is Ash1-dependent, kmt5-dependent and overlaps H4K20me3. But in the right panel, it is not so clear. Part of the WT H3K36me3 appears Ash1 and Kmt5-dependent, although a section is clearly not, while the whole section contain H4K20me3. I cannot find anywhere in the paper how often H3K36me3 and H4K20me3 overlap, how this relates to the chromatin designation, and how it relates to the H3K36me3-dependence on H4K20me3. Does H4K20me3 occur with non-Ash1 H3K36me3 when it is outside of defined facultative

heterochromatin? This is directly relevant, for instance, to the discussion 388-392. In other fungi, Ash1-H3K36me3 is not associated with transcription.

H4K20me3 and H3K36me3 overlap in both, euchromatin and facultative heterochromatin. We made this clearer in the "ChIP-seq uncovers different heterochromatin states in *Z. tritici*" paragraph (line 204ff). We furthermore added analyses how many regions with Set2- and Ash1-mediated H3K36me3 overlap with H4K20me3. While >98% of Ash1-mediated H3K36me3 overlaps with H4K20me3, ~80% of presumably Set2-mediated H3K36me3 overlaps with H4K20me3. The regions that lose H3K36me3 in the *kmt5* deletion strain are Ash1-dependent, which suggests different mechanisms of H4K20me3 deposition in- versus outside of facultative heterochromatin. We have added the bed files containing peaks for H3K27me3, H3K36me3 and H4K20me3 in wild type, Δ*kmt5* and Δ*ash1*, as well as the Set2-dependent and Ash1-dependent H3K36me3 peaks to the zenodo submission.

6. Related to the previous suggestion, kmt5 and ash1 KOs have similar numbers of DEG, but the overlap of regulated transcripts is ~<50%. Do the authors think this point is reflected in the title of the paper- 'H3K20me3 controls Ash1 and transcriptional silencing'. There is not a direct analysis for ash1KO DEG that reports how many of these genes overlaps H4K20me3 and reside in H3K36me3 regions dependent on kmt5. If the other 50% of ash1 KO transcripts are independent of H4K20me3/Kmt5 it seems that point needs to be made more clear.

We agree with the reviewer that the transcriptional responses in the mutant are not that clear and not all DEGs observed in the *ash1* deletion strain are shared with those observed in the *kmt5* deletion strain. We do report the number of DEGs in facultative heterochromatin for each mutant in the text (line 314ff), which may not be the most accurate assessment since many genes are incorrectly annotated in these regions. Hence, the new annotation of transcripts coming from facultative heterochromatin. As H4K20me3 occurs outside of Ash1-mediated H3K36me3 regions and Kmt5 is also likely responsible for H4K20me1/2, there are many additional factors that influence the transcriptome in the Δ*kmt5* deletion strain. As mentioned in the response above, it is difficult to determine the exact Ash1-dependent H3K36me3 regions, as dynamics may change and Set2-dependent H3K36me3 may move into formerly Ash1-repressed regions. We also changed the title of the manuscript to tone down the impact of Kmt5 and H4K20me3.

Minor:

1. Line 135-136 "[…] and more likely general genome stability." Why would this data point to general genome stability? The effects are quite specific to specific chromosomes of the accessory chromosomes checked. Maybe the statement is true, but I don't see how the presented data leads to this as a logical conclusion.

We agree with the reviewer that this statement is speculative and removed it.

2. It is hard to know what the significance is of the increased transcription and newly annotated

genes on the accessory in kmt5KO. It is interesting, I support its inclusion in the paper, but the text could be much shorter. The observations are interesting, but incomplete, and may contribute to the previous reviewer negative comments. There is very little text in the discussion regarding these findings. I suggest shortening the results text, lines 280-348.

We agree with this statement and modified, and shortened this part of the results section. Like the reviewer, we also feel that we should report our results, even though the discussion is terse. One reviewer suggested to add to this section, so we tried our best to find a compromise.

Reviewer #4: This manuscript focuses on the role of H4K20me3 and Ash1-mediated H3K36me3 in facultative heterochromatin in the important wheat pathogen Zymoseptoria tritici. The authors identify the lysine methyltransfersases (KMTases) responsible for H4K20me- and heterochromatin-associated H3K36me (Kmt5 and Ash1 respectively) and show that loss of these KMTases results in increased loss of accessory chromosomes. Using ChIP-seq they map the genome-wide distribution of H4K20me3 and show that Kmt5 is required for the proper distribution of H3K27me3 and Ash1 mediated H3K36me. Importantly, H4K20me is required for silencing and deletion of kmt5 results in the de-repression of genes located in facultative heterochromatin. They also demonstrate that the Z. tritici Eaf3/MRG15 homologue is required for Ash1 activity at H4K20me-enriched regions. Overall, this is a high-quality study that is important for several reasons. The function of H4K20me in fungi is very poorly understood and as the authors point out, genome-wide analyses of H4K20me distribution have not been performed even in model fungal systems. Furthermore, there is little known about the interplay between H4K20me3 and H3K27me marks and also this study explains why the loss of H3K23me3 alone has only a minor effect on transcriptional activation from facultative heterochromatin in Z. tritici. I have a just few minor points that the authors may want to consider.

We would like to thank Reviewer 4 for the positive review of and comments on our manuscript.

1) Were multiple isolates of the different (Δkmt5, Δash1, Δeaf1, K20M etc) mutants generated and examined? There can be variability in the phenotypes associated with different isolates. Although I am certainly not suggesting that experiments need to be repeated with multiple independent isolates, it would be good to know that an initial characterisation revealed consistent phenotypes.

Yes, we have multiple transformants for most transformations (listed in table S4; for two transformations we only obtained one correct transformant but for all other mutants we used two independent transformants). Initially, we used two biological replicates (independent transformants) for the ChIP-seq experiments and found that they always were very similar. When repeating ChIP-seq experiment (mostly for Δ*kmt5*, as we included this one as a control in several experiments), we used one of these transformants. Table S4 lists the independent transformants for each strain and we added an extra column to table S5 detailing which

transformant was used for each sequencing experiment.

2) The corresponding author has demonstrated that H3K27me3 promotes chromosome instability in Z. tritici. Do the authors anticipate that the increased accessory chromosome loss associated with Δkmt5 and Δash1 is dependent upon Kmt6/H3K27me3?

Based on the available data, we can only speculate whether there is a connection between H3K27me3 and the chromosome losses we observed in *kmt5* and *ash1* mutants. The accessory chromosomes that are lost at very high rates (14, 16 in *kmt5* and 16 in *ash1*) show slightly more H3K27me3 enrichment than in wild type (S10 Fig), which may thus contribute to the instability. We also observed overall more transcriptional activity from accessory chromosomes, which could be another factor. However, we do not yet have a clear answer as to which other factors contribute to accessory chromosome loss in these mutants.

3) The authors report that loss of set2 leads to severe growth defects. It would be useful to have a supplementary figure of the Δset2 mutant phenotype.

We have added a new supplementary figure (S9 Fig) that shows growth on plates and a microscopy image of the Δ*set2* mutant. Overall, the *set2* mutants germinate much more slowly, they form a pin-point colony after circa two weeks, and attain the size as shown in the figure after 4-6 weeks. Under the microscope, the strains are immediately filamentous, rather than forming more rapidly dividing cells, as WT or our other mutants do. Even though we have now tried multiple times, we have not been able to accumulate sufficient tissue for ChIP analyses.

4) What is the impact of deletion of kmt5, ash1, etc on virulence? Given the hypothesis that genes in facultative heterochromatin are differentially expressed during host colonisation (e.g. Miele et al. 2020 mBio), I am surprised that wheat infection assays with these mutants were not included.

Although *Z. tritici* is a plant pathogen, the aim of this study is not to address any effects on pathogenicity. Based on the *in vitro* assays and transcriptional changes we fully expect changes in the ability to infect wheat, but since multiple HMTs are involved in regulating the expression of many genes, some of which are virulence-associated, we do not think that wheat infection assays would add any meaningful insights to the current study.

5) Loss of kmt5 and ash1 results in novel transcripts from accessory chromosomes. Are these mutants also associated with an increased level of cryptic intragenic transcripts from bona fide genes on essential/core chromosomes?

This is an interesting suggestion, but at the moment we are not able to include a comprehensive analysis here. Our current data may not be sufficient to address this topic with any level of certainty.

6) Is it possible to show a composite plot of histone modifications for genes marked by H4K20me3, H3K27me3 and H3K36me?

We are not sure what exactly the reviewer is suggesting here but hope that the additional analyses on histone mark enrichment, peak calling, and additional figures are sufficient to address this.

7) Typo: Lines 456 and 457: italicise Δkmt5 and Δash1

Thanks for noticing this! Done!
